# In Solution Identification of the Lysine–Cysteine Redox Switch with a NOS Bridge in Transaldolase by Sulfur K-Edge X-ray Absorption Spectroscopy

**DOI:** 10.1021/acs.jpclett.4c00484

**Published:** 2024-04-12

**Authors:** Ashish Tamhankar, Marie Wensien, Sergio A. V. Jannuzzi, Sayanti Chatterjee, Benedikt Lassalle-Kaiser, Kai Tittmann, Serena DeBeer

**Affiliations:** † Max Planck Institute for Chemical Energy Conversion, Stiftstraße 34-36, 45470 Mülheim an der Ruhr, Germany; ‡ Department of Molecular Enzymology, Göttingen Center of Molecular Biosciences, Georg-August University Göttingen, Julia-Lermonotowa-Weg 3, 37077 Göttingen, Germany; § Max Planck Institute for Multidisciplinary Sciences Göttingen, 37075 Göttingen, Germany; ∥ Synchrotron SOLEIL, L’Orme des Merisiers, Départementale 128, 91190 Saint-Aubin, France; ⊥ Department of Chemistry, Indian Institute of Technology Roorkee, Roorkee, 247667 Uttarakhand, India

## Abstract

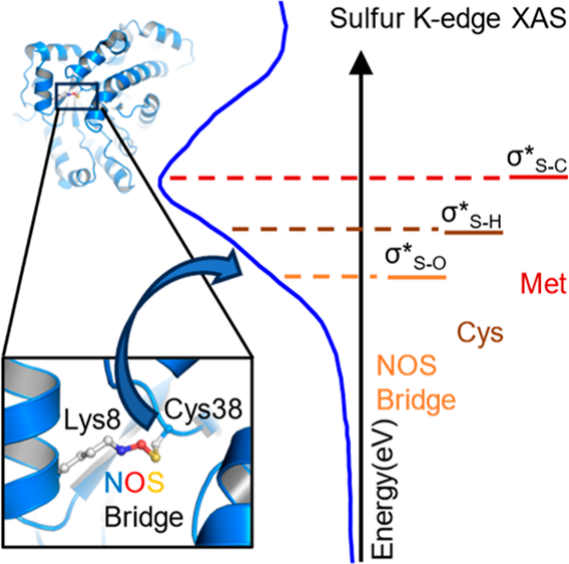

A novel covalent post-translational modification (lysine–NOS–cysteine) was discovered in proteins, initially in the enzyme transaldolase of *Neisseria gonorrhoeae* (*Ng*TAL) [*Nature***2021**, *593*, 460–464], acting as a redox switch. The identification of this novel linkage in solution was unprecedented until now. We present detection of the NOS redox switch in solution using sulfur K-edge X-ray absorption spectroscopy (XAS). The oxidized *Ng*TAL spectrum shows a distinct shoulder on the low-energy side of the rising edge, corresponding to a dipole-allowed transition from the sulfur 1s core to the unoccupied σ* orbital of the S–O group in the NOS bridge. This feature is absent in the XAS spectrum of reduced *Ng*TAL, where Lys-NOS-Cys is absent. Our experimental and calculated XAS data support the presence of a NOS bridge in solution, thus potentially facilitating future studies on enzyme activity regulation mediated by the NOS redox switches, drug discovery, biocatalytic applications, and protein design.

Reactive oxygen species (ROS) play a crucial role in redox signaling and tightly control the biological activity of proteins.^1,2^ ROS controls cell growth, development, metabolism, aging, and response to stress conditions.^1−4^ Elevated levels of ROS induce oxidative stress, which is linked to various pathologies including cancer, neurodegenerative diseases, inflammation, and autoimmune conditions.^5,6^ Major chemical modifications of cysteine residues in redox-sensitive proteins have been linked to the underlying mechanisms of redox signaling and oxidative stress. These modifications include glutathionylation, nitrosylation, and the formation of disulfide bridges.^7,8^

Recently, a novel covalent post-translational linkage between amino acids was discovered in the enzyme transaldolase from *Neisseria gonorrhoeae*, the gonorrhea-causing pathogen. The intramolecular linkage is a Lys–NOS–Cys bridge formed by oxidizing the amine side group of a lysine and the thiol of a cysteine residue and acts as an allosteric redox switch (Figure 1). The proteins’ oxidized and reduced X-ray crystallographic structures suggested a loaded-spring mechanism with a structural relaxation upon redox activation that propagates from the regulatory allosteric lysine–cysteine redox switch site at the protein surface to the active site at the protein interior. This causes a reconfiguration of key catalytic residues evoking an increase in enzymatic activity by several orders of magnitude.^9^ Further studies have identified the NOS switch in crystal structures across a variety of systems and organisms including SARS-CoV-2, potentially playing an important role in regulation, cellular defense, and replication.^10,11^ Moreover, computational insights have also been laid out on exploratory reaction mechanisms of the formation of the NOS bridge.^12^

**Figure 1:**
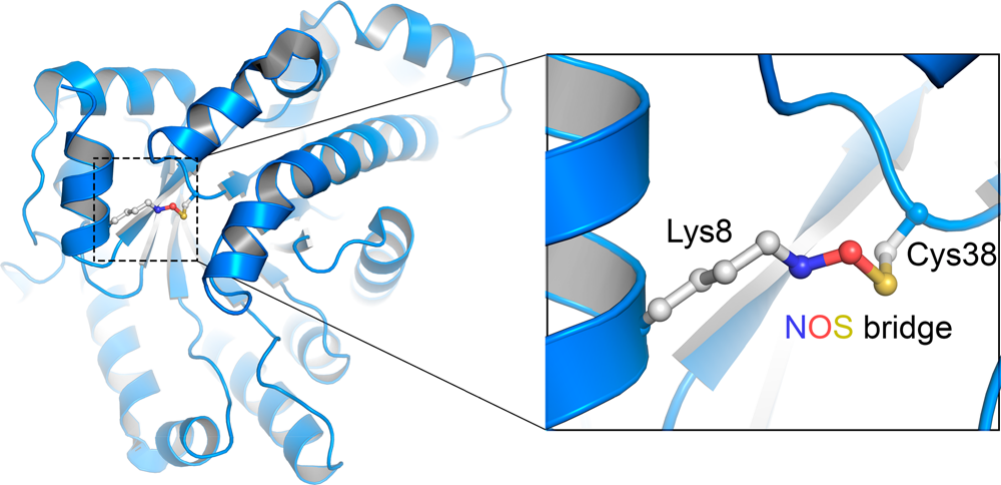
Allosteric Lys–NOS–Cys redox bridge site in the oxidized state of the transaldolase enzyme of *Neisseria gonorrhoeae* (PDB ID: 6ZX4).

Even though the NOS bridge’s chemical identity was clearly shown by protein crystallography at sub-ångstrom resolution, its presence in solution has so far never been directly demonstrated. Previous studies with mass spectrometry suggested that the NOS bridge dissolves following the proteolytic processing of the protein prior to the measurements.^9^ Very recent mass spectrometry analysis revealed that NOS cross-links might be detectable if the protein is not proteolytically digested, albeit the chemical identity can only be inferred from additional structural information.^13^ However, the solid-state techniques (X-ray diffraction and X-ray crystallography) are highly limited in analyzing dynamic systems like proteins. X-ray crystallography may trap proteins in specific conformations, potentially masking their dynamic behavior. In order to avoid structural changes in the protein and bypass the limitations of solid-state techniques, we approached this problem on the complete and natively folded protein to detect the NOS bridge. Sulfur K-edge X-ray absorption spectroscopy (XAS) has already demonstrated its capacity to investigate the sulfur functional groups by providing sulfur-specific speciation information on the absorbing sulfur. The near edge region of sulfur K-edge XAS is dominated by the dipole allowed transition of 1s electrons to unoccupied molecular orbitals with significant sulfur *n*p character. Due to its relatively narrow line widths and wide energy shift range^14^ throughout a range of oxidation states, sulfur exhibits exceptionally informative near-edge spectra. This method has been used to investigate metal–ligand covalency in transition metal complexes^15−17^ and a wide range of metalloenzymes.^18−21^ S K-edge XAS has also been used to determine sulfur speciation in intact biological samples, showing clear differences between methionine, cysteine, and oxidized glutathione with a disulfide bond.^22−24^

In this study, we present the first in-solution detection of an NOS bridge using S K-edge XAS. A comparison of the data collected from the oxidized form of the protein (Ox-*Ng*TAL), the reduced form (Red-*Ng*TAL), as well as the Cys38Ser (Cysteine38Serine) mutant (the NOS linkage cannot form in this variant) under similar conditions establishes the spectral shifts due to changes in the local sulfur environment and provides direct experimental evidence for the presence of the NOS bridge in Ox-*Ng*TAL in solution, which had previously only been established crystallographically. These experimental observations are supported by a systematic time-dependent density functional theory (TDDFT) study and computational analyses of the S K-edge XAS.^25^ The in-solution detection of the NOS redox switch presented herein relies on an element specific technique not requiring crystallization, thus enabling higher throughput analysis of enzyme activity regulation under physiologically relevant conditions.

The sulfur K-edges in *Ng*TAL samples are consistent with the presence of cysteine and methionine^22,24^ indicated by contributions at the S K-edge peak at 2472.7 eV (Figure 2). A distinctive shoulder on the rising edge of Ox-*Ng*TAL is observed at 2471.1 eV in addition to the main peak at 2472.7 eV. In Red-*Ng*TAL, however, the shoulder is absent, and only the main peak is observed at 2472.7 eV. The low energy shoulder on the rising edge in Ox-*Ng*TAL cannot be attributed to sulfur oxidations, since such oxidations are well established to shift the edge to higher energy.^26^ Rather, the presence of the lower energy shoulder on the rising edge is most consistent with an S(1s) to σ* transition, where σ* is the antibonding orbital formed between the S(Cys) and the O in the NOS bridge. This assignment is further supported by the fact that the shoulder on the rising edge is absent in the Cys38Ser mutant, in which the NOS bridge cannot form. Our experiments do also exclude the presence of a disulfide bridge in line with the structural and functional findings (disulfide exhibit two distinct peaks, see ref (20)). We also note that the Ox-*Ng*TAL also shows an additional peak beyond the rising edge at 2473.5 eV, which is absent in Red-*Ng*TAL and the Cys38Ser mutant.

**Figure 2:**
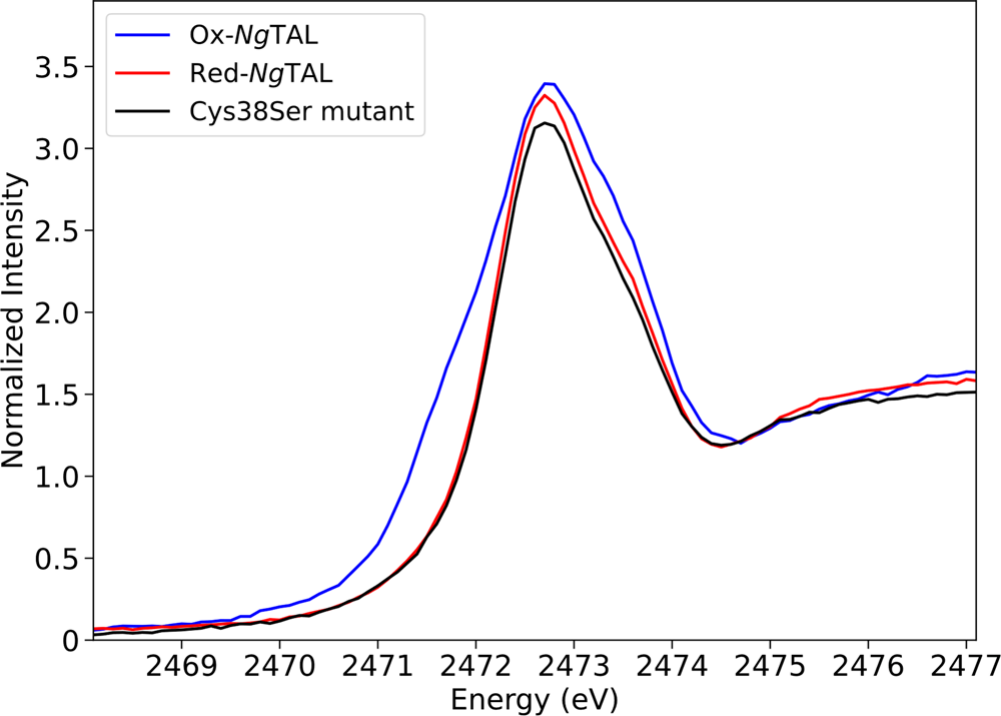
Experimental S K-edge XAS spectra of the oxidized state (Ox-*Ng*TAL, blue), reduced state (Red-*Ng*TAL, red), and the Cys38Ser mutant (black) of the enzyme *Ng*TAL. All spectra were normalized to 1.0 in the postedge region.

The experimental spectra correspond to an average of all seven sulfur sites found in the *Ng*TAL crystal structure: three cysteine residues (Cys38, Cys87, and Cys90) and four methionine residues (Met1, Met32, Met78, and Met136) (Figures S12–S14). However, only one sulfur, in Cys38, is involved in the NOS bridge.^9^ To understand the spectral transformations, we first examined the allosteric redox switch site Lys8–Cys38 minimal cluster models (Figure S1) using TDDFT K-edge XAS calculations. The calculated spectra for the minimal models of Ox-*Ng*TAL and Red-*Ng*TAL are shown in Figure S2. Although the calculated spectra are distinct from the experimental ones (as expected since not all sulfurs in the protein are included in the minimal model), we note that the calculated and experimental difference spectra (in green) are in good agreement. This suggests that the primary transformation that occurs upon reduction of Ox-*Ng*TAL is the loss of the NOS bridge. This simplified model also allows us to rigorously assign the isolated transitions involved in the NOS bridge transition. In the Ox-*Ng*TAL minimal model, the main edge transition is dominated by a single intense transition at 2472.1 eV from the S(1s) to the LUMO, whose main character is σ*_SO_ between the sulfur of Cys38 and the oxygen atom forming the NOS bridge (Figure 3A). The second and third transitions (Figure S3) are from S(1s) to the σ*_ON_ and σ*_SC_ molecular orbitals involving the bridge O–N bond and the S–C bond of Cys38, respectively. In contrast, in the Red-*Ng*TAL minimal model, the primary edge transition at 2472.6 eV is due to a 1s to σ*_SHC_ transition to the unoccupied antibonding molecular orbital delocalized over the S–H–C atoms of Cys38 (Figure 3B). The transition at 2473.3 eV is from S(1s) to σ*_SC_ (Figure S4).

**Figure 3:**
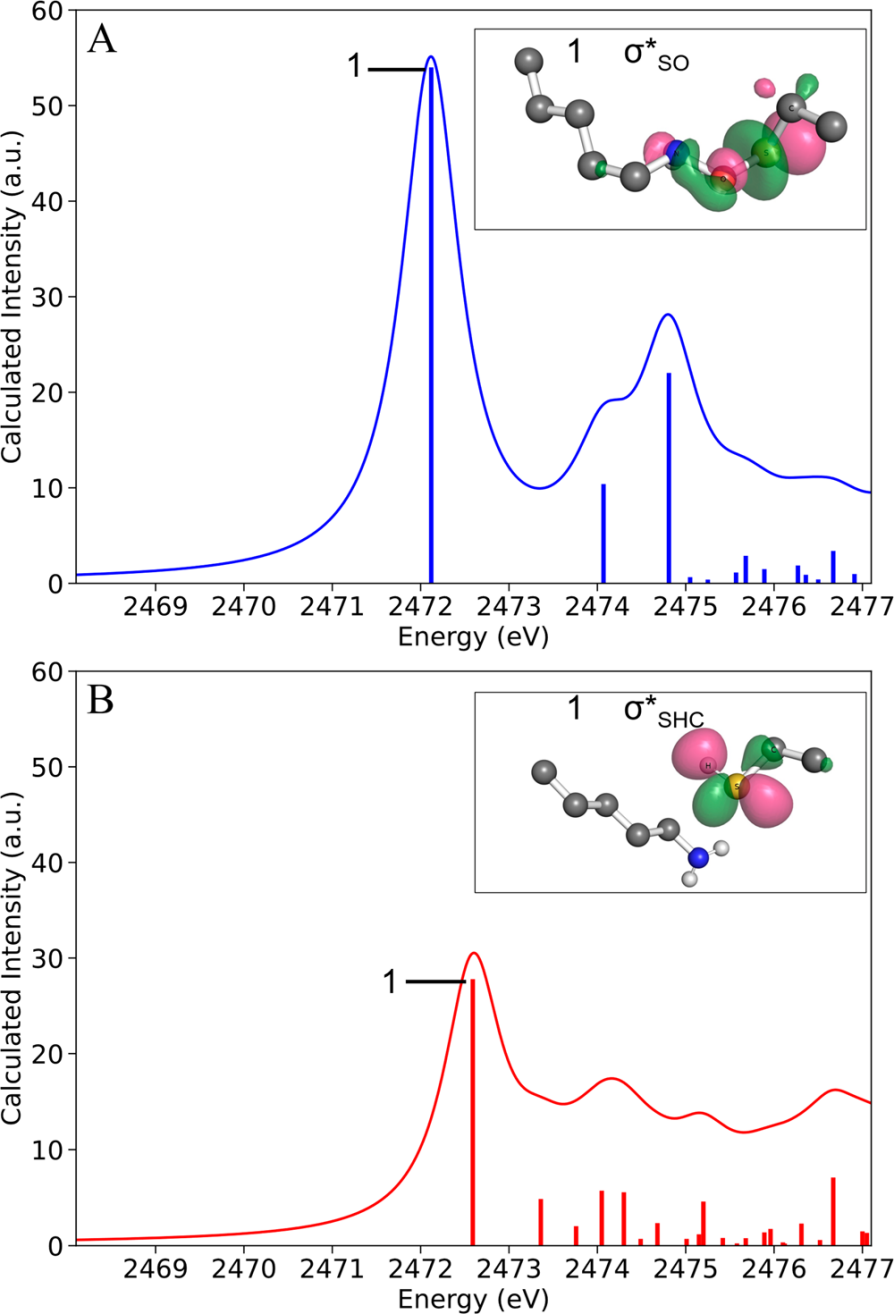
Calculated spectra along with molecular orbitals of the main transitions for the minimal models of (A) oxidized and (B) reduced *Ng*TAL (Lys/Cys site, Figure S1).

In order to more effectively model the experimental spectra and to take the protein environment into consideration, in the next step, we included the cluster models of all seven sulfur sites based on the *Ng*TAL crystal structure in our calculated spectra. The cumulative spectra of Ox-*Ng*TAL Red-*Ng*TAL considering all seven sulfur sites (Figures S5 and S6) resulted in calculated S K-edge XAS spectra that very reasonably reproduce the trends in the experimental spectra (Figure 4). The calculated difference spectrum (cumulative Ox-*Ng*TAL minus cumulative Red-*Ng*TAL; dashed green line) is also in good agreement with the experimental difference data (Ox-*Ng*TAL minus Red-*Ng*TAL; solid green line) for the cumulative plots, which lends credence to the interpretation at this level of theory. We note that there are some differences in the cumulative TDDFT vs experimental spectrum, particularly for the Ox-*Ng*TAL above 2473.2 eV. This can be attributed to the well-known limitations of TDDFT in properly describing high energy Rydberg states.^27^ The issues at high energy, however, largely cancel in the difference spectra and highlight the fact that the low energy portion of the spectrum can be rigorously correlated to a molecular orbital-based picture.

**Figure 4:**
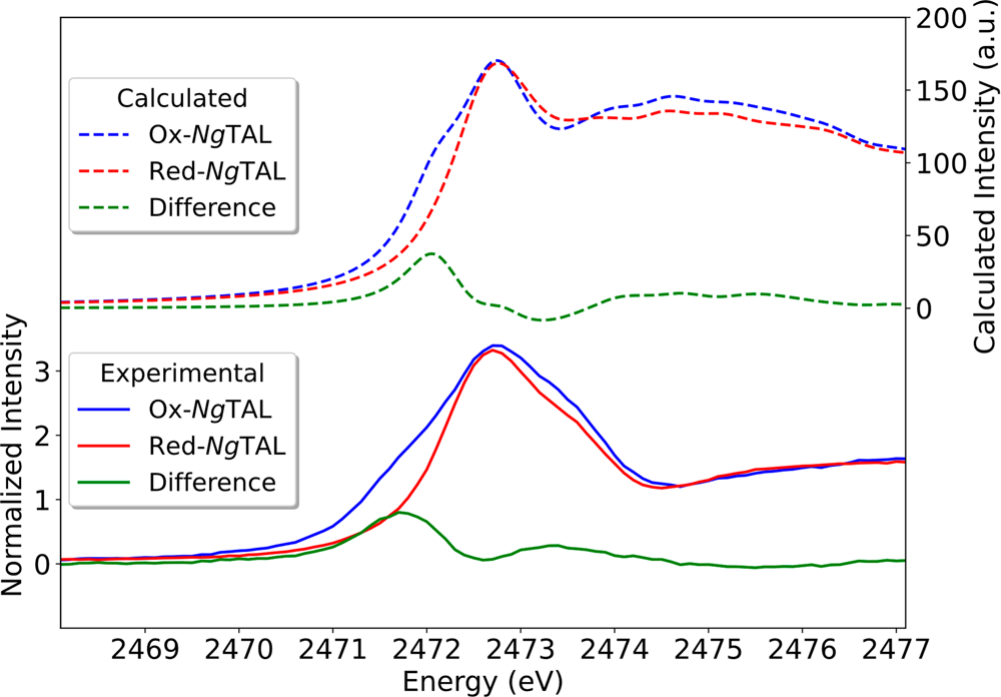
Sulfur K-edge experimental XAS (solid lines) vs cumulative calculated spectra by TDDFT (dashed lines). Difference spectra between Ox-*Ng*TAL minus Red-*Ng*TAL (solid/dashed green). Calculated cumulative Ox-*Ng*TAL spectrum shows the NOS bridge feature on the rising edge with a shift to lower energy (dashed blue), while the cumulatively calculated spectrum for Red-*Ng*TAL shows the usual K-edge characteristic of cysteine and methionine at ∼2472.1 eV (dashed red).

Mutation of Cys38 to Ser did not result in the appearance of a low energy shoulder, in either the experimental or calculated spectra in this case. The cumulative spectra for the Cys38Ser mutant also depicted the cysteine and methionine contributions for each sulfur site (Figure S7) in the protein. We note that the Met1 residue was unresolved in the crystal structure of the Cys38Ser mutant and therefore not modeled in the crystallographic refinement.^9^ However, the intensity of the cumulative calculated S K-edge XAS spectra for the Cys38Ser mutant relative to Red-*Ng*TAL is in better agreement with the experimental data when Met1 residue contributions from oxidized or reduced *Ng*TAL models were incorporated (Figure S11), suggesting the presence of Met1 in the Cys38Ser mutant.

Further insight into the factors that modulate the S K-edge XAS spectra can be obtained from examining the calculated spectra not just for the cumulative addition of different sulfur sites but also for each individual sulfur site. The contributions of individual sulfur sites to the total spectra are shown in Figures S8, S9, and S10 (see the SI). These computed spectra highlight that the average K-edge energy shifts by +0.2 to +0.4 eV to higher energy for the methionine residues relative to the average value for cysteine residues in all three *Ng*TAL samples (Table S1 in the SI). This shift to higher energy is attributed to the energetically higher σ*_SC_ unoccupied molecular orbital formed in the case of methionine (R-S-CH_3_) relative to σ*_SH_ in the case of cysteine and is in full agreement with previous experimental observations.^24^ Further, examination of the contributions of individual cysteine residues shows modulations on the order of ±0.05 eV relative to the average Cys K-edge energy for all three protein models. This highlights the influence that neighboring residues included in the cluster model have on the resultant calculated S K-edge XAS spectra. We note, however, that the largest modulation of −0.5 eV relative to the Cys average K-edge is observed in the presence of the S–O bond in Ox-*Ng*TAL (Lys8-NOS-Cys38), indicating that this is a robust indicator of the presence or absence of a NOS bridge.

In conclusion, the experimental sulfur K-edge XAS and TDDFT calculations provide a clear and straightforward means to detect the NOS redox switch in solution. The establishment of the shoulder at 2471.1 eV as a spectroscopic marker in S K-edge XAS for in-solution detection should facilitate novel avenues in drug discovery, biocatalytic applications, and protein design, with further biological implications in the context of redox signaling, oxidative stress, and many human disease states.

## Data Availability

The data that support the findings of this study are openly available on Edmond open data repository of the Max Planck Society at 10.17617/3.BLQZ5X

## Supporting Information Available

Detailed information about experimental procedures, additional figures as mentioned in the text and coordinates of optimized structures (PDF)
