# Supplementary material for: In Solution Identification of the Lysine–Cysteine Redox Switch with a NOS Bridge in Transaldolase by Sulfur K-Edge X-ray Absorption Spectroscopy
Source: J Phys Chem Lett. 2024 Apr 12;15(16):4263–7. doi: 10.1021/acs.jpclett.4c00484 (PMC11056971; doi:10.1021/acs.jpclett.4c00484)
Supplement: Supplementary file 1 — jz4c00484_si_001.pdf [file jz4c00484_si_001.pdf]

Supplementary Information for

In Solution Identification of the Lysine-Cysteine Redox Switch with a NOS Bridge in Transaldolase by Sulfur K-edge X-ray Absorption Spectroscopy

Ashish Tamhankar[1], Marie Wensien[2,3], Sergio A. V. Jannuzzi[1], Sayanti Chatterjee[1,†], Benedikt Lassalle-Kaiser[5], Kai Tittmann[2,3*], Serena DeBeer[1*]

[1]Max Planck Institute for Chemical Energy Conversion, Stiftstraße 34-36, 45470 Mülheim an der Ruhr, Germany.

[2]Department of Molecular Enzymology, Göttingen Center of Molecular Biosciences, Georg-August University Göttingen, Julia-Lermonotowa-Weg 3, 37077 Göttingen, Germany.

[3]Max Planck Institute for Multidisciplinary Sciences Göttingen, 37075 Göttingen, Germany.

[†]Department of Chemistry, Indian Institute of Technology, Roorkee, Roorkee, 247667 Uttarakhand, India.

[5] Synchrotron SOLEIL, L'Orme des Merisiers, Départementale 128, 91190 Saint-Aubin, France.

*Correspondence to: kai.tittmann@biologie.uni-goettingen.de, serena.debeer@cec.mpg.de

Contents:

1. List of Supplementary Figures pg. S2
2. Experimental Methods pg. S3
3. Supplementary Figures (S1-S17) pg. S5
4. Supplementary Table (S1) pg. S22
5. Supplementary References pg. S23
6. Sample Input for Hydrogen Geometry Optimization pg. S25
7. Sample Input for TDDFT S K-edge XAS pg. S26
8. Optimized Atomic Coordinates for Oxidized *Ng*TAL minimal model pg. S27
9. Optimized Atomic Coordinates for Reduced *Ng*TAL minimal model pg. S28
10. Optimized Atomic Coordinates for Oxidized *Ng*TAL pg. S29
11. Optimized Atomic Coordinates for Reduced *Ng*TAL pg. S43
12. Optimized Atomic Coordinates for Cys38Ser *Ng*TAL pg. S57

1. List of Supplementary Figures

Figure S1. Hydrogen optimized minimal cluster models of the oxidized and reduced *Ng*TAL.
Figure S2. Sulfur K-edge experimental vs TDDFT calculated spectra of the Lys-NOS-Cys site (minimal model).
Figure S3. TDDFT spectra along with molecular orbitals of the transition states for the minimal models of oxidized *Ng*TAL (Lys/Cys site).
Figure S4. TDDFT spectra along with molecular orbitals of the transition states for the minimal models of reduced *Ng*TAL (Lys/Cys site).
Figure S5. Experimental vs calculated cumulative TDDFT XAS for oxidized *Ng*TAL.
Figure S6. Experimental vs calculated cumulative TDDFT XAS for reduced *Ng*TAL.
Figure S7. Experimental vs calculated cumulative TDDFT XAS for Cys38Ser *Ng*TAL.
Figure S8. Experimental vs calculated single sulfur center TDDFT XAS for oxidised *Ng*TAL.
Figure S9. Experimental vs calculated single sulfur center TDDFT XAS for reduced *Ng*TAL.
Figure S10. Experimental vs calculated single sulfur center TDDFT XAS for Cys38Ser *Ng*TAL.
Figure S11. Experimental vs calculated cumulative TDDFT XAS for Cys38Ser *Ng*TAL with and without Met1 residue.
Figure S12. Hydrogen optimized cluster models of Oxidized *Ng*TAL.
Figure S13. Hydrogen optimized cluster models of Reduced *Ng*TAL.
Figure S14. Hydrogen optimized cluster models of Cys38Ser *Ng*TAL.
Figure S15. Multiple scans of oxidized *Ng*TAL sample.
Figure S16. Multiple scans of reduced *Ng*TAL sample.
Figure S17. Multiple scans of Cys38Ser *Ng*TAL sample.

2. Experimental Methods

Protein sample preparation

The oxidized form of *Neisseria gonorrhoeae* transaldolase (*Ng*TAL) wild-type (Ox-*Ng*TAL), the reduced form of the wild-type enzyme (Red-*Ng*TAL), as well as the Cys38Ser variant (cannot form the NOS crosslink) were recombinantly expressed and purified as previously described.[1] The cells of custom-made small volume copper sample holders (prepared by the machine shop of the Max Planck Institute for Chemical Energy Conversion as well as the Synchrotron SOLEIL) were loaded with 20 µL of 2.19 mM Ox-*Ng*TAL, 2.16 mM Red-*Ng*TAL supplemented with 10 mM TCEP (tris(2-carboxyethyl)phosphine) and 2.16 mM Cys38Ser, respectively. The proteins were loaded as duplicates. All proteins were prepared freshly prior to the measurements and kept in 50 mM GlyGly pH 8.0 buffer. The sample cells were sealed with sulfur-free foil and frozen in liquid nitrogen.

S K-edge XAS measurements

XAS data were measured at the LUCIA beamline[2] at the SOLEIL synchrotron, as previously reported,[3] with a ring current of 500 mA and a nominal energy of 2.3 GeV. A Si(111) double-crystal monochromator was utilized for selection of the incident energy. The incident beam energy was calibrated for sulfur by setting the first maximum of the spectrum of $Na_2S_2O_3\cdot5\ H_2O$ to 2472.0 eV. A spot size of ~2 x 4 mm was utilized at the sample and the estimated flux was 5.5 x 10^11 Ph/s. Samples were set in a measurement chamber under vacuum (10^{-6} mbar) and maintained in a He-cooled cryostat at 100 K during measurements. Samples were maintained at 100 K rather than liquid He temperature due to limited availability of He, and to keep the temperature constant throughout the experiment. Data were measured in fluorescence yield utilizing a Bruker SDD detector windowed at the sulfur Kα emission, placed at a 90° angle with respect to the incident beam and 30° outgoing angle with respect to the sample. Spectra were monitored for radiation damage throughout the course of data collection, with 0.1 eV step-size for the monochromator scan in the pre-edge range. For all samples, multiple spots were measured to ensure damage free averages. The presented data represent the average of 3-4 10 minute scans, which showed no evidence of significant damage (see Figures S15-S17). Data were processed and analyzed utilizing the Demeter package.[4] All spectra were normalized to 1.0 in the post-edge region.

Computational methods

Model preparation

The cluster models for QM calculations were prepared using the X-ray crystal structures of Ox-*Ng*TAL, Red-*Ng*TAL and Cys38Ser mutant (PDB id: 6zx4, 6zwf, 7bbw respectively).[1] The protonation states of the amino acids were carefully checked using Propka 3.[5,6] Protonation assignments were performed at pH 8, which is the same pH value as the buffer used to prepare the samples. The minimal models of the Lys-NOS-Cys bridge site consisted of Lys8 and Cys38 residues (Fig. S1). The cluster model of the Lys-NOS-Cys bridge site consisted of the residues Lys8, Cys38, Ile14, Trp15, Gln13, Thr101, Thr97, Glu93 (Fig. S12A). For the other sulfur centers in the proteins, 2 cysteines and 4 methionines smaller cluster models were constructed (Fig. S12, S13, S14). Cys87, the cluster model consisted of Cys87, Asp88, Asn134, Ala135, Val83, Ile130. The Cys90, consisted of Cys90, Leu33, Leu29, Val104, Val40, Asn134. While Met1 consisted of Met1, Ile294, Gln35. Met32 consisted of Met32, Leu23, Leu29, Leu16. Met78 consisted of Met78, Leu350, Val61, Phe51. Met136 consisted of Met136, Val197, Gln195, Phe103. In all cluster models the amino acids were terminated at their Cα positions and substituted with hydrogens to form methyl groups.

All calculations in this study were performed using ORCA 5.0.4 software package.[7–12] The protocol used in this study for geometry optimization and TDDFT XAS K-edge calculations were based on ref. 13, and updated to the current ORCA version.

Geometry Optimization

The hydrogen atoms in the cluster model were optimized using BP86[14] functional with ZORA[15] relativistic approximation using ZORA-def2-TZVP(-f)[16] basis set. The SARC/J decontracted auxiliary basis set[17] was implemented. In addition to the auxiliary basis, the chain of spheres approximation (RIJCOSX)[18] was used for the RI approximation to the coulomb integrals. Atom-pairwise dispersion with correction based on tight binding partial charges (D4)[19,20] was implemented. The calculations were performed using CPCM(water)[21] implicit solvation with "TightOPT" and "SlowConv" settings turned on. A denser integration grid !DEFGRID3 was employed. The optimized structures were used to perform XAS K-edge calculations using Time-dependent Density Functional Theory (TDDFT).

TDDFT XAS calculation

X-ray absorption spectra were calculated using TDDFT with BP86[14] functional with the Tamm-Dancoff[22] approximation. Scalar relativistic effects were included using ZORA[15] with ZORA-def2-TZVP(-f)[16] basis set. The SARC/J[17] was used as the auxiliary basis for coulomb fitting. In addition to the auxiliary basis the chain of spheres approximation (RIJCOSX)[18] was used for the RI approximation to the coulomb integrals. The calculations were performed using water implicit solvation as per CPCM[21] with default settings. The SCF convergence criteria were set with "TightSCF" and "SlowConv" keywords.

The absorption spectra were plotted using the orca_mapspc utility tool with broadening of 0.8 eV with Voigt line fitting with uniform energy shift of 59.4 eV applied to compare with the experimental data. The cumulative absorption spectra were plotted by adding each sulfur center contribution with the same weighting. The contributions from the electric dipole, electric quadrupole, and magnetic dipole were added to determine the oscillator strength. The transition attributions were based on canonical molecular orbitals generated using orca_plot utility tool and analyzed in Pymol version 2.5.4.[23]

3. Supplementary Figures

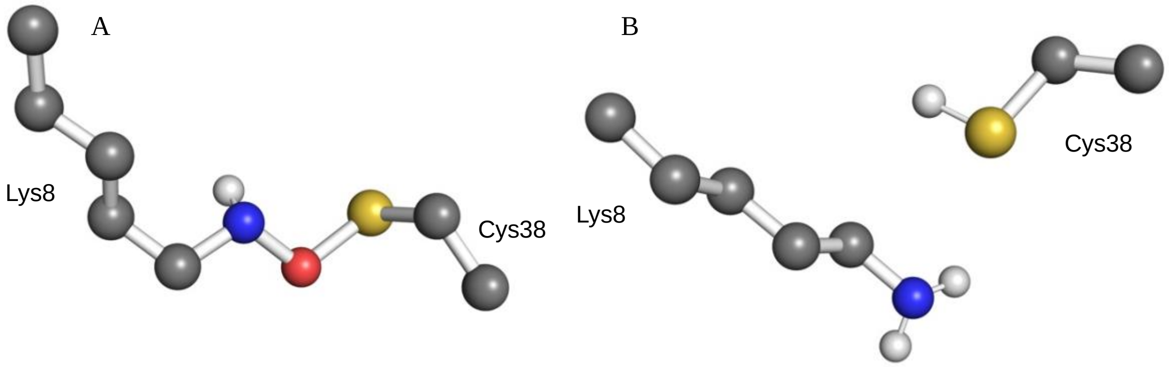

Figure S1. Hydrogen optimized minimal cluster models of the oxidized and reduced *Ng*TAL. A) Minimal model of the Ox-*Ng*TAL consisting of Lys8 and Cys38. B) Minimal model of the Red-*Ng*TAL consisting of Lys8 and Cys38.

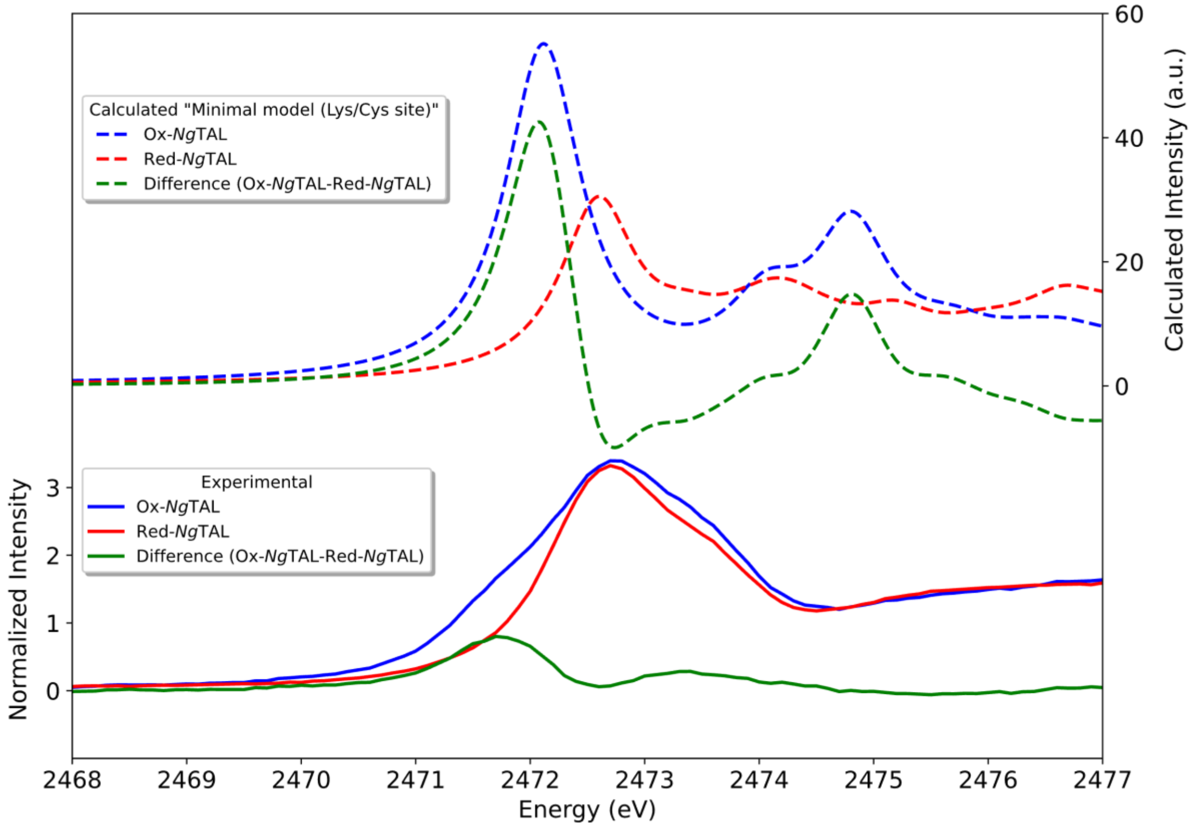

Figure S2. Sulfur K-edge experimental XAS vs TDDFT calculated spectra of the Lys/Cys site (minimal model). The experimental oxidized and reduced transaldolase are shown in solid blue and red respectively. The calculated Ox-*Ng*TAL (including only the Lys/Cys site) shows intense K-edge peak with a shift to lower energy (dashed blue), while the Red-*Ng*TAL (including only the Lys/Cys site) shows K-edge at ~2472.3 eV with much lower intensity (dashed red). The oxidized and reduced *Ng*TAL samples show inaccurate edges compared to the experimental data. The calculated difference spectrum (dashed green) is in the qualitatively correct trend but fails to capture the experimental difference spectrum (green) intensities.

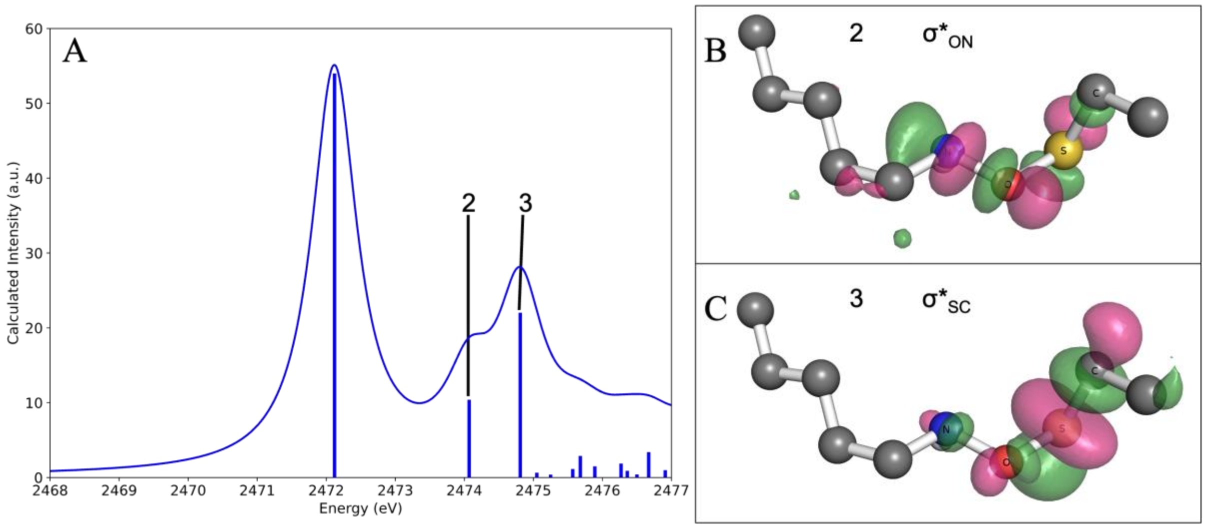

Figure S3. TDDFT spectrum along with the molecular orbitals populated after the indicated electronic transitions for the minimal models of oxidized *Ng*TAL (Lys/Cys site). A) TDDFT spectra for the Ox-*Ng*TAL Lys/Cys site. B) The transition at 2474.1 eV (2) is to the σ* antibonding orbital of the oxygen atom of the NOS bridge and nitrogen of Lys38 (N-S). C) The following transition at 2474.8 eV (3) is to the σ* antibonding orbital formed by the C-S bond of Cys38.

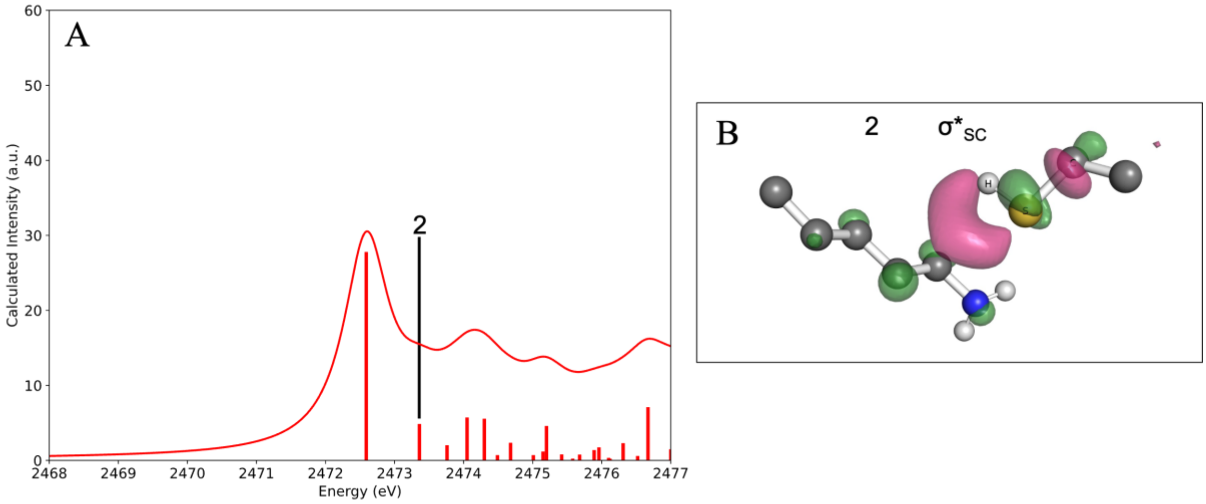

Figure S4. TDDFT spectrum along with the molecular orbital occupied after the indicated electronic transition for the minimal models of reduced *Ng*TAL (Lys/Cys site). A) TDDFT spectra for the Red-*Ng*TAL Lys/Cys site. B) The transition at 2473.3 eV (2) is 1s to σ* transition to the unoccupied molecular orbital formed by C-S bond of Cys38.

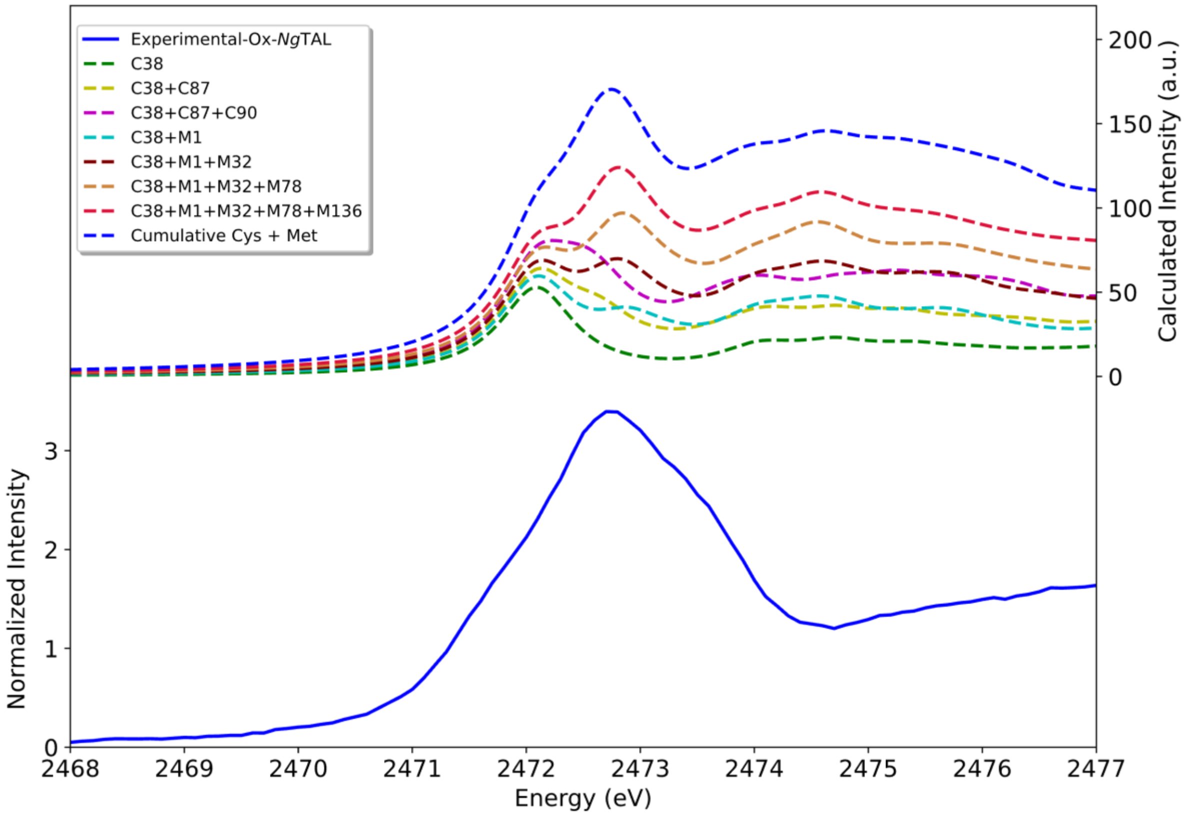

Figure S5. Experimental vs calculated cumulative TDDFT XAS for oxidized *Ng*TAL. Experimental Ox-*Ng*TAL (solid blue) along with the cumulative calculated TDDFT of Ox-*Ng*TAL (dashed blue). The calculated data is shown by adding each sulfur center (Cys/Met) to the NOS site (Cys38) data. The legend describes each amino acid residue number that is added to the spectra. The cumulative spectra with cysteine and methionine residues accurately compares with the experimental spectra of the Ox-*Ng*TAL with accurate reproduction of the shift of K-edge to lower energy.

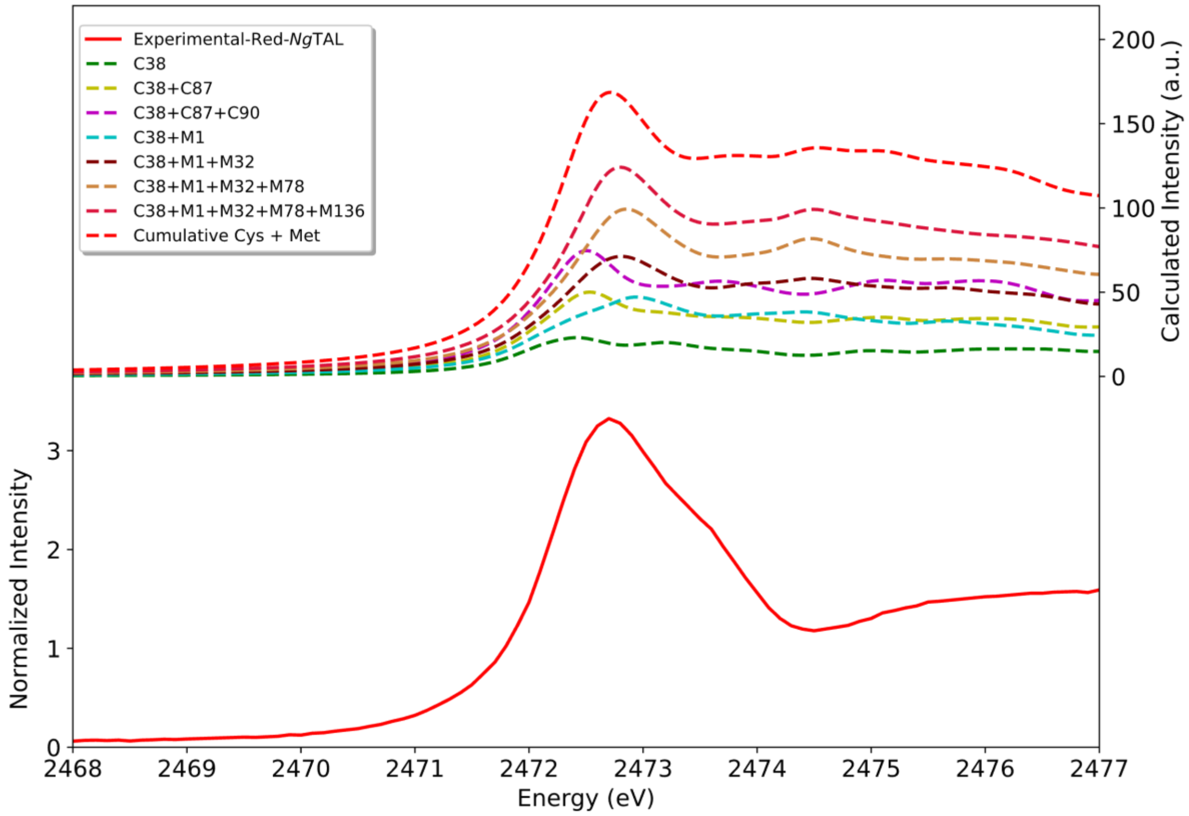

Figure S6. Experimental vs calculated cumulative TDDFT XAS for Red-*Ng*TAL. Experimental Red-*Ng*TAL (solid red) along with the cumulative calculated TDDFT of Red-*Ng*TAL (dashed red). The calculated data is shown by adding each sulfur center (Cys/Met) to the Cys38 site data. The legend describes each amino acid residue number that is added to the spectra. The cumulative spectra with cysteine and methionine residues accurately compare with the Red-*Ng*TAL experimental spectra.

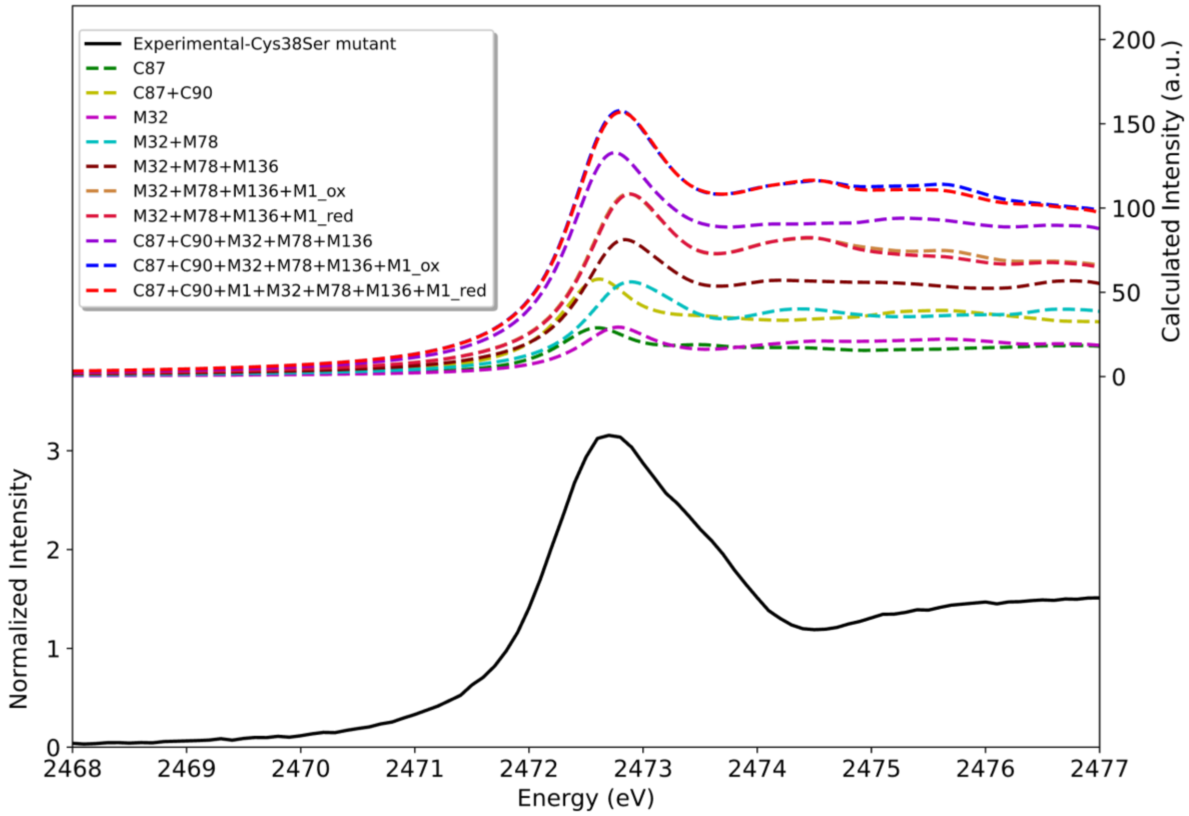

Figure S7. Experimental vs calculated cumulative TDDFT XAS for Cys38Ser *Ng*TAL. Experimental *Ng*TAL Cys38Ser mutant (solid black) along with the cumulative calculated TDDFT *Ng*TAL of the Cys38Ser mutant (dashed red and dashed blue). The calculated data is shown by adding each sulfur center (Cys-/Met) to the Cys38 site data. The legend describes each amino acid residue number that is added to the spectra. The Met1 residue was unresolved in the Cys38Ser mutant and not modelled in the pdb file, therefore the cumulative spectra were generated with adding Met1 spectra from Ox-*Ng*TAL (dashed blue) and from Red-*Ng*TAL (dashed red). The cumulative spectra with cysteine and methionine residues accurately compares with the experimental spectra of the Cys38Ser mutant of *Ng*TAL.

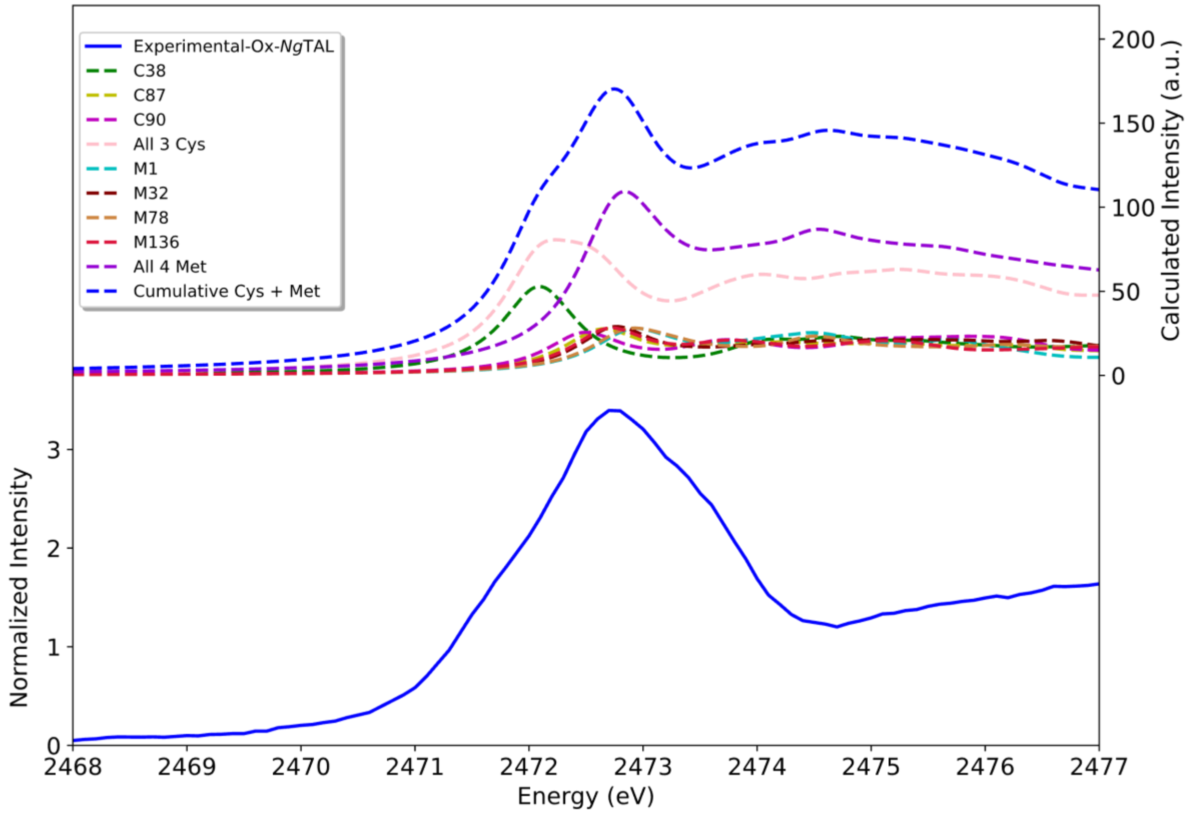

Figure S8. Experimental vs calculated single sulfur center TDDFT XAS for oxidised *Ng*TAL. Experimental XAS spectra of the Ox-*Ng*TAL with calculated TDDFT spectra of each sulfur center plotted individually. The K-edge of the Cys38 is shifted to lower energy significantly with higher intensity, while the rest of the cysteine sulfur K-edges are at characteristic cysteine sulfur K-edges.

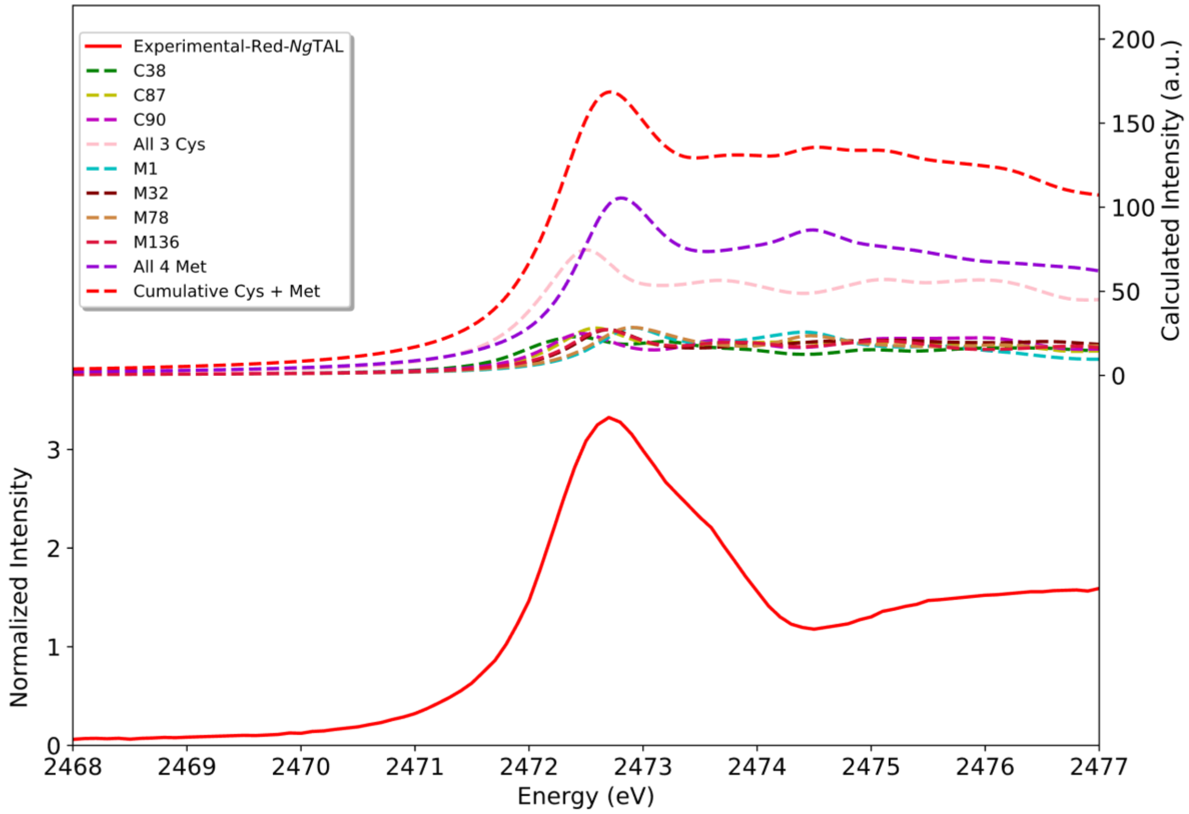

Figure S9. Experimental vs calculated single sulfur center TDDFT XAS for reduced *Ng*TAL. Experimental XAS spectra of the Red-*Ng*TAL with calculated TDDFT spectra of each sulfur center plotted individually. The K-edge of the Cys38 is not shifted to lower energy. All sulfur K-edges are comparable to cysteine and methionine K-edges.

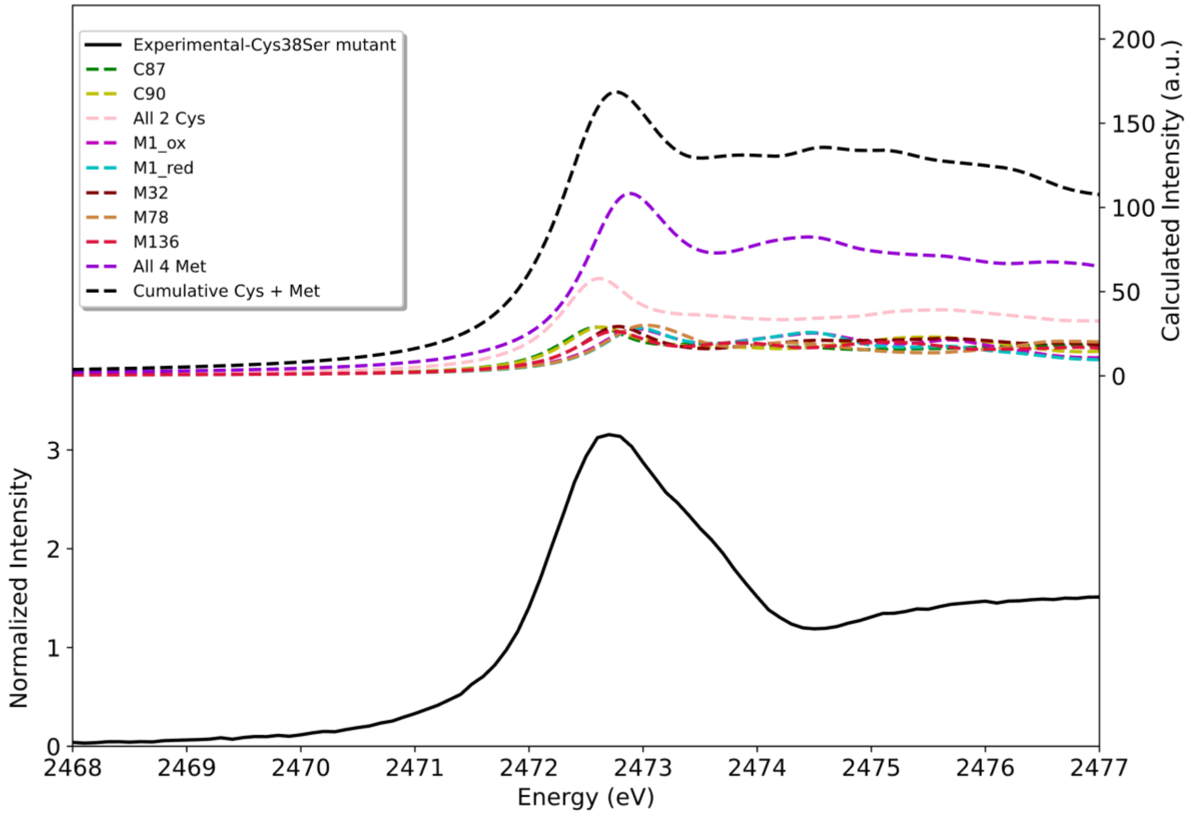

Figure S10. Experimental vs calculated single sulfur center TDDFT XAS for Cys38Ser *Ng*TAL. Experimental XAS spectra of the Cys38Ser mutant *Ng*TAL with calculated TDDFT spectra of each sulfur center plotted individually. The Met1 residue was unresolved in the Cys38Ser mutant and not modelled in the crystal structure, therefore the single sulfur center spectra of Met1 from Ox-*Ng*TAL (M1_ox, dashed magenta) and from Red-*Ng*TAL (M1_red, dashed cyan) were used for the Cys38Ser mutant.

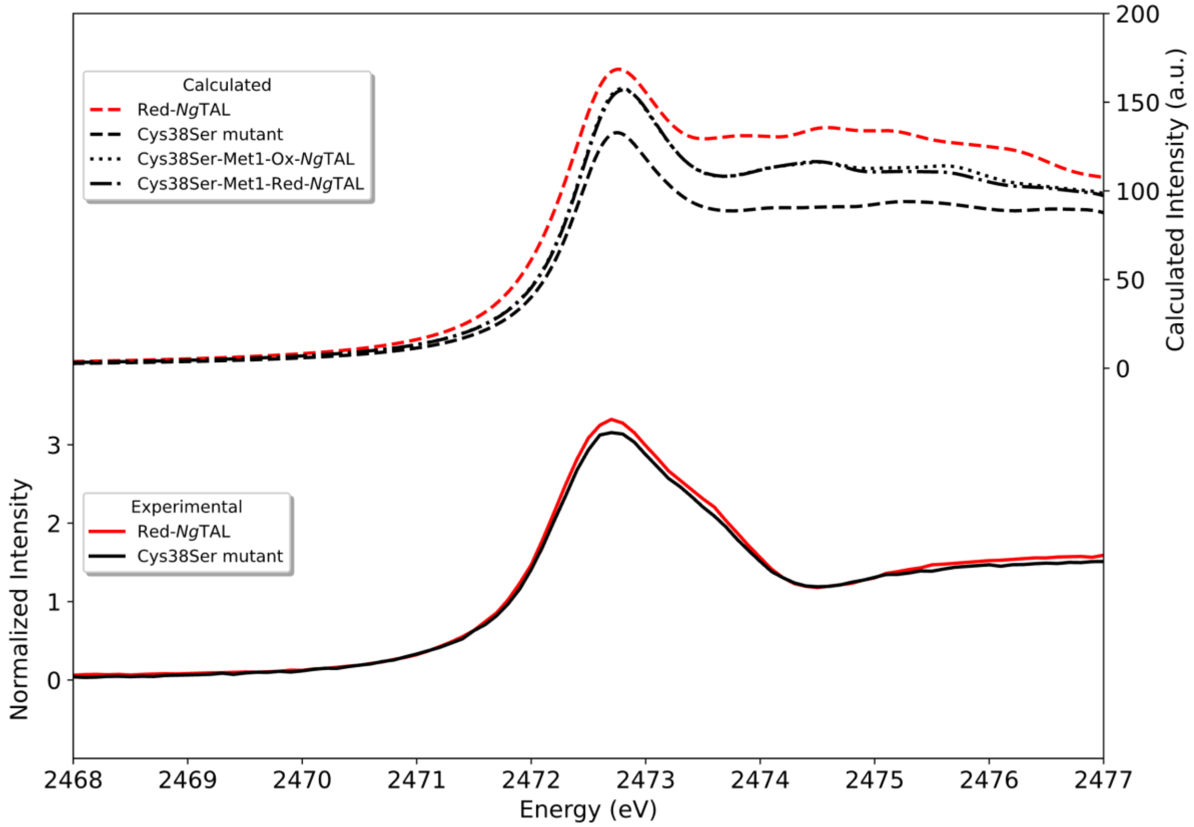

Figure S11. Experimental vs calculated cumulative TDDFT XAS for Cys38Ser *Ng*TAL with and without Met1 residue. Experimental *Ng*TAL Cys38Ser mutant (solid black) along with the cumulative calculated TDDFT *Ng*TAL of the Cys38Ser mutant without Met1 residue (dashed black). The Met1 residue was unresolved in the Cys38Ser mutant and not modelled in the pdb file, therefore the cumulative spectra were generated with adding Met1 spectra from Ox-*Ng*TAL (dotted black) and from Red-*Ng*TAL (dashdot black). The experimental data for Red-*Ng*TAL (solid black) and calculated Red-*Ng*TAL are shown to better compare the relative intensities with Cys38Ser mutant. The cumulative spectra with Met1 residue incorporated accurately compares with the experimental spectra of the Cys38Ser mutant of *Ng*TAL.

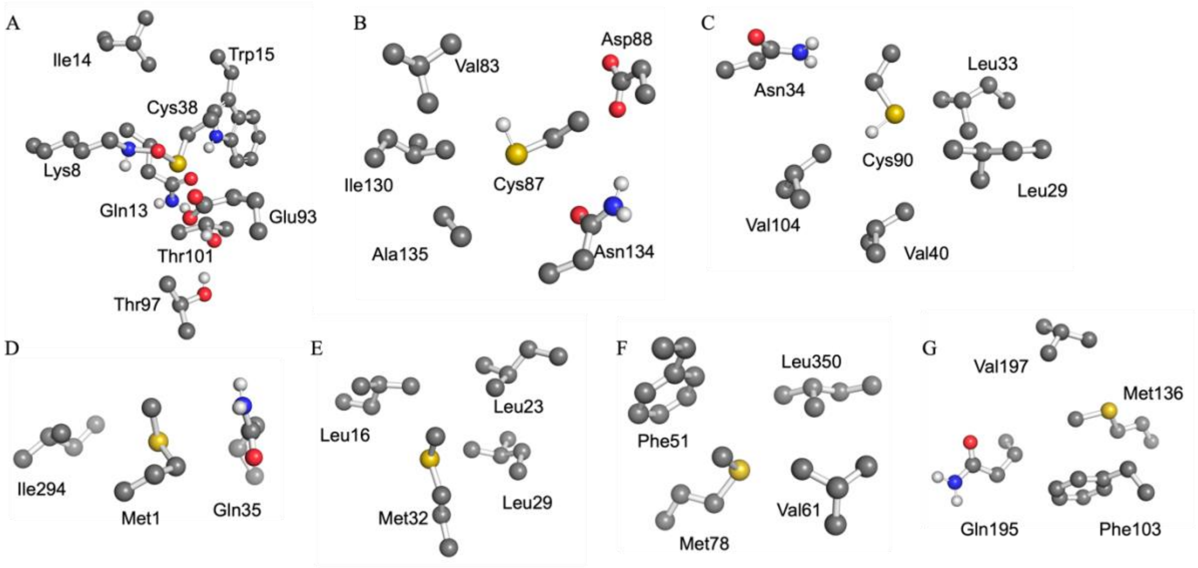

Figure S12. Hydrogen optimized cluster models of oxidized *Ng*TAL. The figure shows hydrogen optimized cluster model for each sulfur centers in the Ox-*Ng*TAL sample. (Only amino acid residues displayed in the figure were considered for optimization). A) Cluster model of NOS bridge cite (Cys38). B) Cluster model of Cys87. C) Cluster model of Cys90. D) Cluster model of Met1. E) Cluster model of Met32. F) Cluster model of Met78. G) Cluster model of Met136. Non-polar hydrogen atoms were omitted for clarity.

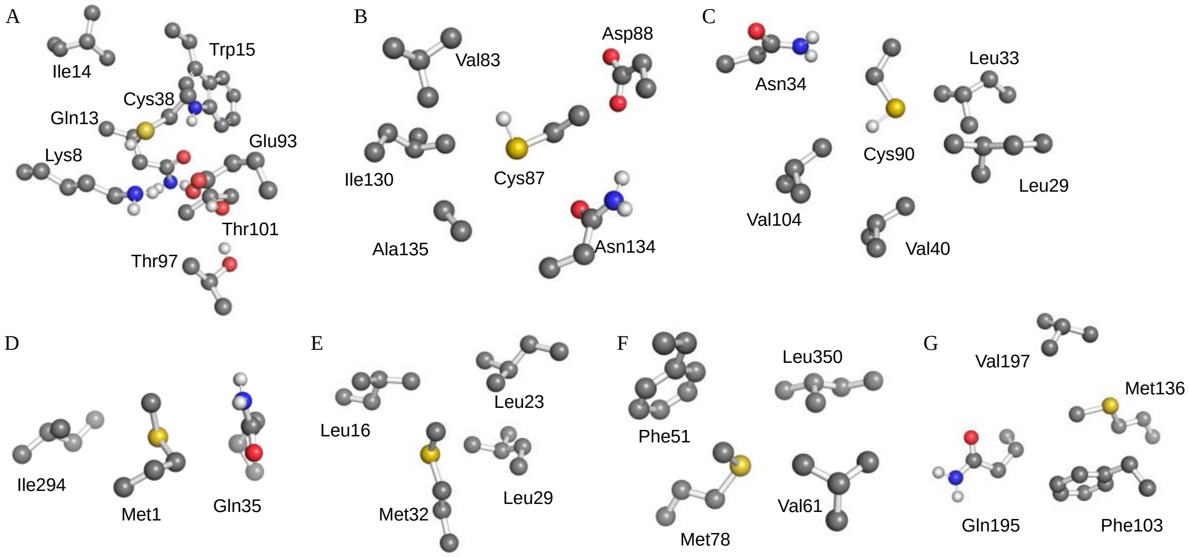

Figure S13. Hydrogen optimized cluster models of reduced *Ng*TAL. The figure shows hydrogen optimized cluster model for each sulfur centers in the Red-*Ng*TAL sample. (Only amino acid residues displayed in the figure were considered for optimization). A) Cluster model of NOS bridge cite (Cys38). B) Cluster model of Cys87. C) Cluster model of Cys90. D) Cluster model of Met1. E) Cluster model of Met32. F) Cluster model of Met78. G) Cluster model of Met136. Non-polar hydrogen atoms were omitted for clarity.

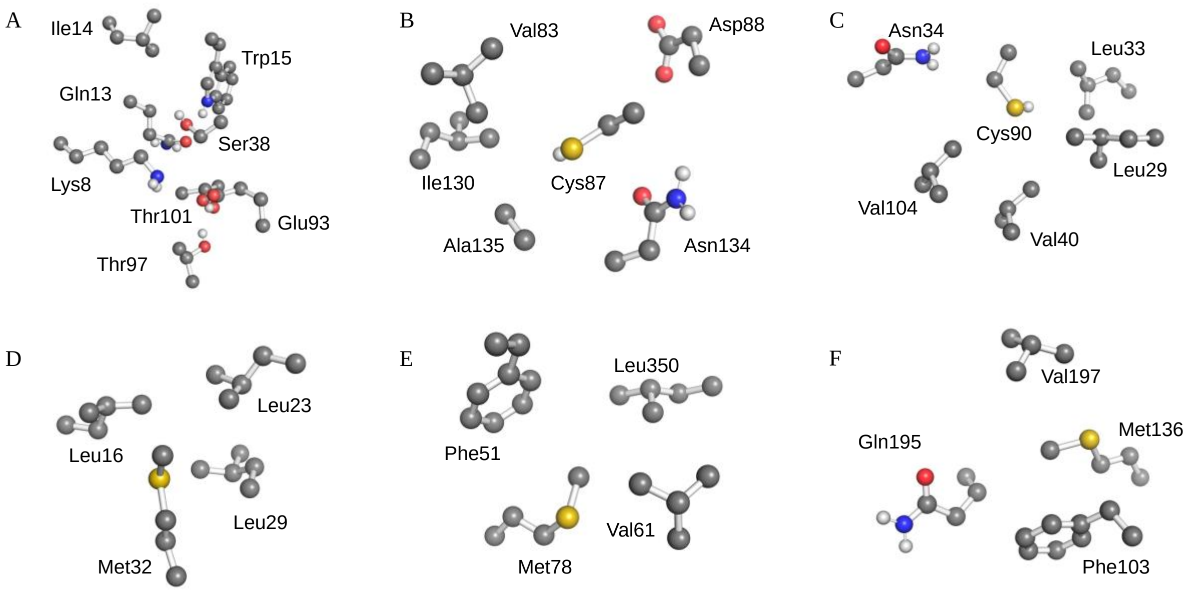

Figure S14. Hydrogen optimized cluster models of Cys38Ser *Ng*TAL. The figure shows hydrogen optimized cluster model for each sulfur centers in the C38S *Ng*TAL sample. (Only amino acid residues displayed in the figure were considered for optimization). A) Cluster model of NOS bridge site (Ser38). B) Cluster model of Cys87. C) Cluster model of Cys90. D) Cluster model of Met32. E) Cluster model of Met78. F) Cluster model of Met136. Non-polar hydrogen atoms were omitted for clarity.

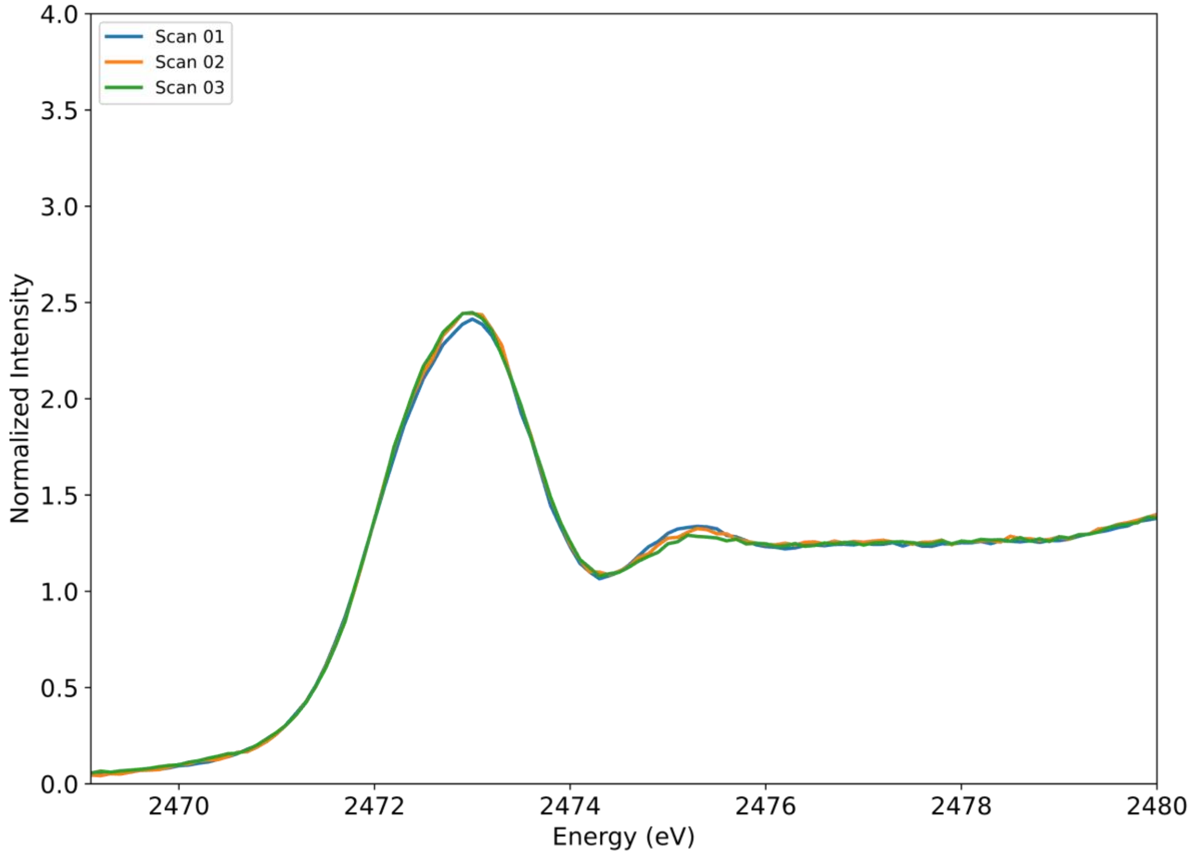

Figure S15. Multiple scans of oxidized *Ng*TAL sample. The figure shows three different scans for the Ox-*Ng*TAL sample before damage was observed. All spectra are normalized to 1.0 in the post-edge region.

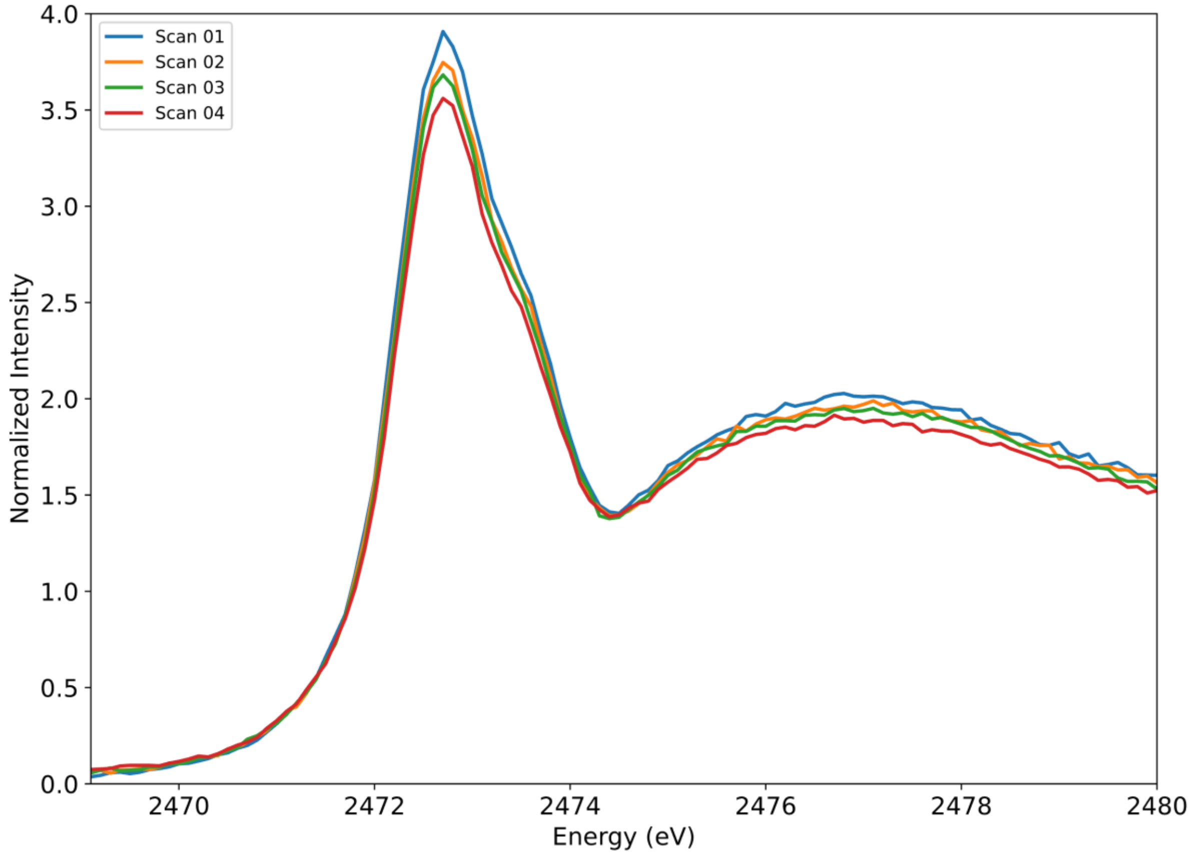

Figure S16. Multiple scans of reduced *Ng*TAL sample. The figure shows four different scans for the Red-*Ng*TAL sample before damage was observed. All spectra are normalized to 1.0 in the post-edge region.

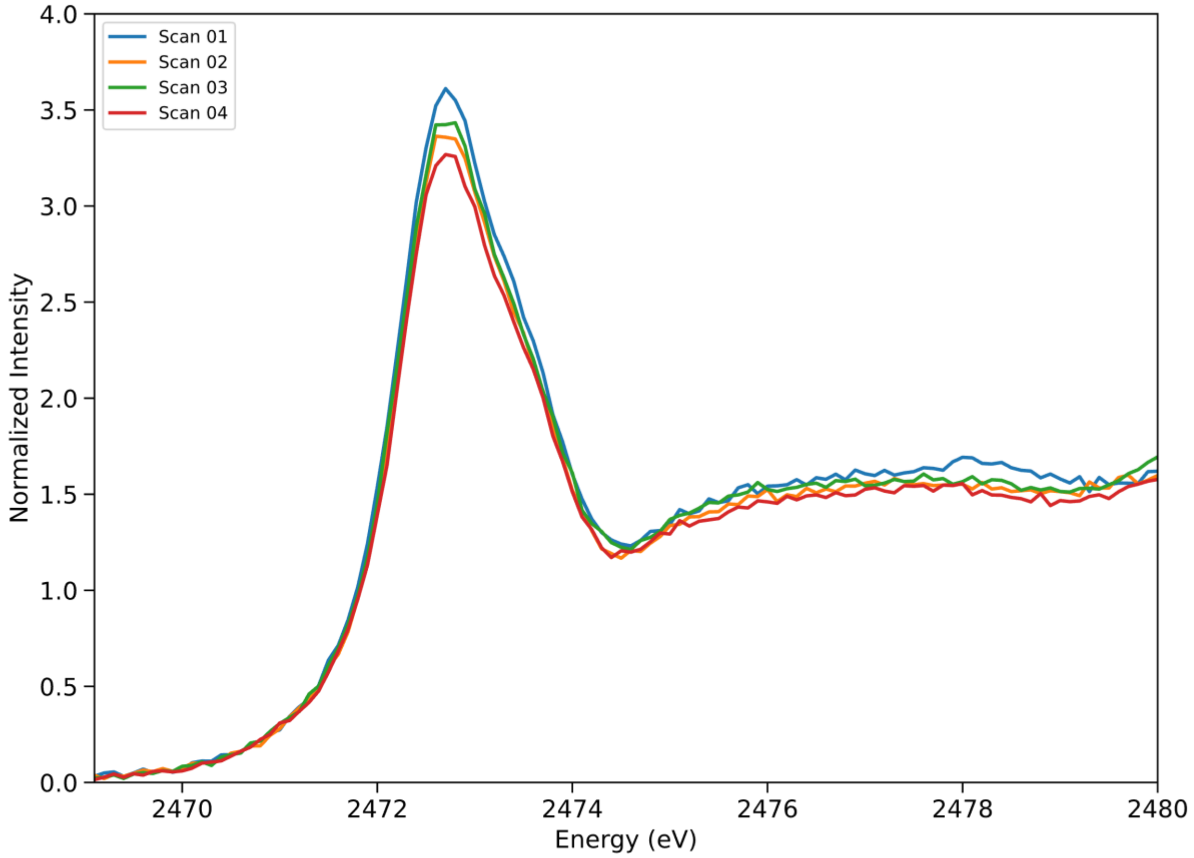

Figure S17. Multiple scans of Cys38Ser *Ng*TAL sample. The figure shows four different scans for the Cys38Ser *Ng*TAL sample before damage was observed. All spectra are normalized to 1.0 in the post-edge region.

Table S1. Sulfur K-edge main peak transition energy (eV) for each sulfur center in the three *Ng*TAL protein samples. All energy values are after applying the uniform shift of 59.4 eV to compare with the experimental data.

Residue	Sulfur K-edge main transition peak energy (eV)		
	Ox-*Ng*TAL	Red-*Ng*TAL	Cys38Ser mutant
Cys38	2472.1	2472.5	-
Cys87	2472.6	2472.6	2472.6
Cys90	2472.5	2472.5	2472.6
Cysteine residue average	2472.55[a]	2472.53[b]	2472.6
Met1	2472.9	2472.9	2472.9[c]
Met32	2472.8	2472.7	2472.7
Met78	2472.9	2472.9	2472.9
Met136	2472.7	2472.7	2472.7
Methionine residue average	2472.82	2472.80	2472.80

[a] Cysteine residue average in the Ox-*Ng*TAL is excluding Cys38.
[b] Cysteine residue average in Red-*Ng*TAL is including Cys38, Cys87 and Cys90.
[c] Met1 value taken from Ox-*Ng*TaL.

4. References

(1) Wensien, M.; Von Pappenheim, F. R.; Funk, L.-M.; Kloskowski, P.; Curth, U.; Diederichsen, U.; Uranga, J.; Ye, J.; Fang, P.; Pan, K.-T.; Urlaub, H.; Mata, R. A.; Sautner, V.; Tittmann, K. A Lysine–Cysteine Redox Switch with an NOS Bridge Regulates Enzyme Function. *Nature* **2021**, *593* (7859), 460–464.

(2) Vantelon, D.; Trcera, N.; Roy, D.; Moreno, T.; Mailly, D.; Guilet, S.; Metchalkov, E.; Delmotte, F.; Lassalle, B.; Lagarde, P.; Flank, A.-M. The LUCIA Beamline at SOLEIL. *J. Synchrotron Radiat.* **2016**, *23* (2), 635–640.

(3) Bjornsson, R.; Delgado-Jaime, M. U.; Lima, F. A.; Sippel, D.; Schlesier, J.; Weyhermüller, T.; Einsle, O.; Neese, F.; DeBeer, S. Molybdenum L-Edge XAS Spectra of MoFe Nitrogenase: Molybdenum L-Edge XAS Spectra of MoFe Nitrogenase. *Z. Für Anorg. Allg. Chem.* **2015**, *641* (1), 65–71.

(4) Ravel, B.; Newville, M. ATHENA, ARTEMIS, HEPHAESTUS: Data Analysis for X-Ray Absorption Spectroscopy Using IFEFFIT. *J. Synchrotron Radiat.* **2005**, *12* (4), 537–541.

(5) Olsson, M. H. M.; Søndergaard, C. R.; Rostkowski, M.; Jensen, J. H. PROPKA3: Consistent Treatment of Internal and Surface Residues in Empirical p K_a Predictions. *J. Chem. Theory Comput.* **2011**, *7* (2), 525–537.

(6) Søndergaard, C. R.; Olsson, M. H. M. Improved Treatment of Ligands and Coupling Effects in Empirical Calculation and Rationalization of pKa Values. *J. Chem. Theory Comput.* **2011**, *7* (7), 2284-2295.

(7) Neese, F. The ORCA Program System. *WIREs Comput. Mol. Sci.* **2012**, *2* (1), 73–78.

(8) Neese, F.; Wennmohs, F.; Becker, U.; Riplinger, C. The ORCA Quantum Chemistry Program Package. *J. Chem. Phys.* **2020**, *152* (22), 224108.

(9) Neese, F. Software Update: The ORCA Program System—Version 5.0. *WIREs Comput. Mol. Sci.* **2022**, *12* (5), e1606.

(10) Valeev, E. F. Libint: A Library for the Evaluation of Molecular Integrals of Many-Body Operators over Gaussian Functions. Version http://libint.valeyev.net/2021.

(11) Lehtola, S.; Steigemann, C.; Oliveira, M. J. T.; Marques, M. A. L. Recent Developments in Libxc — A Comprehensive Library of Functionals for Density Functional Theory. *SoftwareX* **2018**, *7*, 1–5.

(12) Ekström, U.; Visscher, L.; Bast, R.; Thorvaldsen, A. J.; Ruud, K. Arbitrary-Order Density Functional Response Theory from Automatic Differentiation. *J. Chem. Theory Comput.* **2010**, *6* (7), 1971–1980.

(13) DeBeer George, S.; Neese, F. Calibration of Scalar Relativistic Density Functional Theory for the Calculation of Sulfur K-Edge X-Ray Absorption Spectra. *Inorg. Chem.* **2010**, *49* (4), 1849–1853.

(14) Becke, A. D. Density-Functional Exchange-Energy Approximation with Correct Asymptotic Behavior. *Phys. Rev. A* **1988**, *38* (6), 3098–3100.

(15) van Lenthe, E.; Snijders, J. G.; Baerends, E. J. The Zero-order Regular Approximation for Relativistic Effects: The Effect of Spin–Orbit Coupling in Closed Shell Molecules. *J. Chem. Phys.* **1996**, *105* (15), 6505–6516.

(16) Weigend, F.; Ahlrichs, R. Balanced Basis Sets of Split Valence, Triple Zeta Valence and Quadruple Zeta Valence Quality for H to Rn: Design and Assessment of Accuracy. *Phys. Chem. Chem. Phys.* **2005**, *7* (18), 3297.

(17) Weigend, F. Accurate Coulomb-Fitting Basis Sets for H to Rn. *Phys. Chem. Chem. Phys.* **2006**, *8* (9), 1057.

(18) Helmich-Paris, B.; de Souza, B.; Neese, F.; Izsák, R. An Improved Chain of Spheres for Exchange Algorithm. *J. Chem. Phys.* **2021**, *155* (10), 104109.

(19) Caldeweyher, E.; Ehlert, S.; Hansen, A.; Neugebauer, H.; Spicher, S.; Bannwarth, C.; Grimme, S. A Generally Applicable Atomic-Charge Dependent London Dispersion Correction. *J. Chem. Phys.* **2019**, *150* (15), 154122.

(20) Caldeweyher, E.; Bannwarth, C.; Grimme, S. Extension of the D3 Dispersion Coefficient Model. *J. Chem. Phys.* **2017**, *147* (3), 034112.

(21) Barone, V.; Cossi, M. Quantum Calculation of Molecular Energies and Energy Gradients in Solution by a Conductor Solvent Model. *J. Phys. Chem. A* **1998**, *102* (11), 1995–2001.
(22) Hirata, S.; Head-Gordon, M. Time-Dependent Density Functional Theory within the Tamm–Dancoff Approximation. *Chem. Phys. Lett.* **1999**, *314* (3–4), 291–299.
(23) Schrödinger, L. *The PyMOL Molecular Graphics System*, Version 2.5.4, 2015.

5. Sample Input for hydrogen geometry optimization.

```
!BP86 TIGHTOPT TIGHTSCF ZORA ZORA-def2-TZVP(-f) Sarc/J D4 CPCM(Water) LargePrint
!DEFGRID3

%pal nprocs 8
   end

%geom
optimizeHydrogens True
end

%output
Print[ P_Basis ] 2
Print[ P_MOs ] 1
Print[P_ReducedOrbPopMO_L] 1
Print[ P_Hirshfeld] 1
end

* xyzfile -1 1 filename.xyz
```

6. Sample input for TDDFT K-Edge XAS.

```
!SP BP86 TIGHTSCF ZORA ZORA-def2-TZVP(-f) Sarc/J D4 CPCM(Water) LargePrint
!DEFGRID3

%Maxcore 3000

%pal nprocs 8
    end
%scf
MaxIter 700
end


%tddft
orbwin[0]= 0,0,-1,-1
doquad true
nroots 300
maxdim 50
DoNTO true
NTOThresh 1e-4
NTOStates 1,2,3,4,5,6,7,8,9,10,11,12
end

%output
Print[ P_Basis ] 2
Print[ P_MOs ] 1
Print[P_ReducedOrbPopMO_L] 1
Print[ P_Hirshfeld] 1
end

* xyzfile -1 1 filename.xyz
```

7. Optimized Atomic Coordinates for Oxidized *Ng*TAL minimal model.

C	-28.419865	25.240183	-13.696468
C	-29.432149	24.103717	-13.562672
C	-28.866434	22.744088	-13.257949
C	-29.969825	21.690002	-13.389344
C	-29.507154	20.274658	-13.123915
N	-28.502575	19.876616	-14.137556
H	-27.701751	25.035972	-14.506038
H	-28.919211	26.193505	-13.922468
H	-27.841735	25.376972	-12.768732
H	-30.175101	24.367657	-12.788829
H	-30.004533	24.039319	-14.505480
H	-28.045237	22.511474	-13.956258
H	-28.434923	22.710859	-12.242820
H	-30.779413	21.913872	-12.675593
H	-30.416698	21.746851	-14.397086
H	-29.048232	20.189662	-12.126059
H	-30.378232	19.592074	-13.150334
H	-28.899989	20.162812	-15.043880
C	-25.628853	16.339158	-14.072081
C	-25.765498	17.725891	-14.765691
S	-27.335684	17.935425	-15.656608
O	-28.454974	18.348603	-14.350253
H	-24.651179	16.261952	-13.573056
H	-26.414777	16.206823	-13.316504
H	-25.709649	15.532740	-14.812599
H	-25.022639	17.853768	-15.567351
H	-25.650881	18.568133	-14.070021

8. Optimized Atomic Coordinates for Reduced *Ng*TAL minimal model.

C	-28.343550	25.031395	-13.672658
C	-29.140852	23.727185	-13.524632
C	-29.257721	22.873659	-14.797542
C	-29.952261	21.514097	-14.541471
C	-29.861376	20.579016	-15.760260
N	-30.568737	19.256575	-15.599166
H	-27.309432	24.828329	-13.991613
H	-28.799043	25.696352	-14.423130
H	-28.301657	25.578696	-12.719847
H	-28.670825	23.111110	-12.738553
H	-30.155228	23.960010	-13.156769
H	-29.803555	23.429922	-15.578546
H	-28.245573	22.685032	-15.198956
H	-29.484293	21.019494	-13.672953
H	-31.011142	21.681066	-14.278289
H	-30.227096	21.129940	-16.649006
H	-28.802094	20.357733	-15.956539
H	-30.192172	18.529893	-16.222159
H	-31.551987	19.377456	-15.854285
C	-25.669301	16.326226	-14.034272
C	-25.795333	17.694184	-14.724112
S	-27.271593	18.587346	-14.279672
H	-25.634848	16.429088	-12.940098
H	-26.510853	15.666773	-14.293581
H	-24.740309	15.843288	-14.366732
H	-24.925005	18.309381	-14.455922
H	-25.770815	17.561187	-15.814304
H	-26.965869	19.732265	-14.929591

9. Optimized Atomic Coordinates for Oxidized *Ng*TAL

Optimized atomic coordinates in Å – oxidized *Ng*TAL Cys38 cluster model

C	-28.41986533032938	25.24018288606792	-13.69646752318529
C	-29.43214860464403	24.10371712937456	-13.56267223751610
C	-28.86643444393502	22.74408806135152	-13.25794894990111
C	-29.96982498993422	21.69000243203532	-13.38934408593279
C	-29.50715355580840	20.27465824403617	-13.12391507369901
N	-28.50257486577036	19.87661586496111	-14.13755550502261
H	-27.70175056571281	25.03597212966515	-14.50603796767184
H	-28.91921069653464	26.19350458156131	-13.92246841614434
H	-27.84173489684909	25.37697191610715	-12.76873213307541
H	-30.17510107004409	24.36765672164770	-12.78882911206323
H	-30.00453281039078	24.03931856508552	-14.50547983866801
H	-28.04523698170720	22.51147446320089	-13.95625786624985
H	-28.43492319167988	22.71085881711315	-12.24282015014780
H	-30.77941313768219	21.91387150417208	-12.67559319628799
H	-30.41669758879505	21.74685124133676	-14.39708603138662
H	-29.04823223127240	20.18966154805138	-12.12605907387930
H	-30.37823178127071	19.59207367953946	-13.15033351220707
H	-28.89998859841116	20.16281248373726	-15.04387963904544
C	-23.92045331402667	21.98636606559931	-15.17067717463619
C	-23.76847768061633	20.90455778254089	-16.24987082602460
C	-24.81777307056810	20.88684748465679	-17.32557503395320
C	-24.51605242152757	19.79759988291032	-18.31238733108551
N	-24.16493442190967	20.18935258785690	-19.52328108119864
O	-24.56695575858021	18.60355329777687	-17.99215764281774
H	-24.80448428411581	21.80532204191579	-14.54079111415829
H	-23.03411128433819	22.01030827770445	-14.52633256821339
H	-24.03004831915607	22.98430120148240	-15.62363749951554
H	-22.77976468550939	21.02699854062248	-16.72570476884955
H	-23.73975724986961	19.92051883920695	-15.75808044317382
H	-25.81010123910884	20.67656256406720	-16.89218392808816
H	-24.87720354954574	21.86532977041756	-17.82651703778694
H	-24.14996212095670	21.16893037144117	-19.78591415904453
H	-23.93276379952667	19.49951405036671	-20.23360177641313
C	-22.23702306280736	20.57633947553493	-12.07210478042506
C	-22.64063456990200	20.98260759617126	-10.64491728086996
C	-22.69403373598545	22.51867433314868	-10.50423062664971
C	-21.69181813194895	20.37760127314379	-9.61980461174217
C	-21.36675626865422	23.23234474329104	-10.67044479531676
H	-21.18469608383615	20.81966192878764	-12.28102842771055
H	-22.36921255995654	19.49665149103465	-12.22053586172538
H	-22.86173912007343	21.08847599043829	-12.81404677132472
H	-23.65876808821588	20.60211510971313	-10.45506925288374
H	-23.41454006488009	22.90989029445843	-11.24305105800166
H	-23.11348175070395	22.76241639629630	-9.51235743147635
H	-21.95101665459297	20.68933003148569	-8.59564487438985
H	-21.72048946827798	19.27724597294137	-9.65118615109769
H	-20.65149128300605	20.68095094502402	-9.81015486281301
H	-21.49825284055563	24.32190148539088	-10.59119869310413
H	-20.63908971942236	22.93676216806844	-9.90064006204411
H	-20.91088011550259	23.03119713238322	-11.65163074878456

C	-20.68667685501767	17.13458669636113	-12.54697474556346
C	-19.93022981304362	16.79833412127211	-13.83821941102100
C	-20.72445170234913	16.99907425260294	-15.11215306821946
C	-22.01298147772819	17.45546354706042	-15.22921346609554
C	-20.26358807128378	16.78161008857368	-16.44709225382067
C	-21.32053223558636	17.10580732397932	-17.31563114832810
C	-19.05428522893623	16.35178707683871	-16.99522408147551
N	-22.37945030694560	17.51412494363070	-16.54912640854646
C	-21.19815639105069	17.04024160232312	-18.69835918207881
C	-18.93089796557576	16.29821343961008	-18.36994106885644
C	-19.99488970589998	16.62941677757858	-19.21249519392567
H	-21.61443948443263	16.54954777070488	-12.46022968615093
H	-20.06489109475814	16.91522110046153	-11.66772398816698
H	-20.95226356638722	18.19727974507148	-12.51338104523881
H	-19.01027657056844	17.40679796375464	-13.88951084550826
H	-19.58138073180941	15.75133557691620	-13.79599693544623
H	-22.70988692452317	17.75872846569157	-14.45371983835248
H	-23.25649775103280	17.89084670408286	-16.93194540357801
H	-18.21201954094159	16.08615554626586	-16.35254807056880
H	-22.03482685515817	17.30891936950622	-19.34628051645599
H	-17.98541935006547	15.97478957133272	-18.81104922528172
H	-19.86100131782374	16.56929572932396	-20.29441110954109
C	-25.62885293688828	16.33915752513938	-14.07208097611356
C	-25.76549756676370	17.72589103803657	-14.76569067429146
S	-27.33568365155682	17.93542470470350	-15.65660834755528
O	-28.45497433091728	18.34860318156693	-14.35025298606013
H	-24.65117940985502	16.26195150614157	-13.57305586587555
H	-26.41477739020153	16.20682262902990	-13.31650414641702
H	-25.70964862718679	15.53273972777520	-14.81259891188925
H	-25.02263884957172	17.85376828471099	-15.56735127251498
H	-25.65088131516503	18.56813256962206	-14.07002053609465
C	-31.20995664100067	12.94837731067116	-17.45922394798079
C	-30.12196827346128	13.19800856882109	-16.42029435934259
C	-29.38004113789590	14.53919760135414	-16.60067451688647
C	-30.28523145430385	15.75246946257692	-16.47734537904172
O	-31.12454673780520	15.76413893800994	-15.56448850158914
O	-30.13872796000957	16.70752628112952	-17.27245958942018
H	-31.98638745561724	13.72737104597978	-17.41469621675422
H	-30.79511414881738	12.94888735797584	-18.47993904780912
H	-31.70150770637175	11.97722913765437	-17.29880234613198
H	-30.56528387628633	13.17697471086121	-15.41249029005882
H	-29.38367581129683	12.37944199075835	-16.45499097115243
H	-28.60321682031970	14.63387338705933	-15.82283392057083
H	-28.85842419985888	14.55557109727166	-17.57005449098823
C	-32.27570938163469	17.30789478643298	-22.53363732235325
C	-31.08420039856214	17.87640667255489	-21.76627441853190
C	-31.42927605626084	18.13350540591121	-20.30801660119846
O	-30.00438724163067	16.93366977249495	-21.85900960383812
H	-33.09999324487839	18.03592635259758	-22.57017891600715
H	-31.99014242455537	17.06144854895167	-23.56698014928030
H	-32.63789032437994	16.39084456209923	-22.04426987558627
H	-30.76862827857686	18.82630103527435	-22.24082797573607
H	-29.56491913388486	16.83402141335665	-20.97166106494875
H	-30.57682824666024	18.55927149302830	-19.76365459100413
H	-32.27064297226943	18.83851714561037	-20.22565799690804

H	-31.71958993797723	17.19384073782175	-19.81439495609151
C	-26.38194167327138	16.48838721223366	-20.13095217226417
C	-27.45776054729829	17.07867090407694	-19.22039177263127
C	-27.71921941246609	18.56849642992402	-19.49326588059073
O	-28.65608688928189	16.32688940626506	-19.46286969783694
H	-25.42941085440625	17.01984803740610	-19.99847036214351
H	-26.22743947576392	15.42465881165416	-19.90097056809446
H	-26.69102359986661	16.57715166274738	-21.18410144052412
H	-27.15820077664844	16.94663751948086	-18.16538190015145
H	-29.25115114221470	16.45924937130551	-18.64040118885372
H	-28.50007630286017	18.95139717808387	-18.82126647341568
H	-26.81147794944385	19.16501268371221	-19.33751427078657
H	-28.05191143468203	18.70528599225504	-20.53292482705662

Optimized atomic coordinates in Å – oxidized *Ng*TAL Cys87 cluster model

C	-18.93578710733907	3.53960528588296	-16.58519951488957
C	-17.85665539065045	3.86380512109875	-17.63123747363823
C	-18.40039395091369	4.77671760429382	-18.71777981225610
C	-16.64420424736723	4.47743039840384	-16.96844598514668
H	-17.55054820355140	2.91300465201984	-18.10501424749767
H	-17.63516937313811	4.99928738177047	-19.47567044127773
H	-19.26118200792712	4.32693797287415	-19.23333113622228
H	-18.73282749183693	5.73659763244561	-18.28780258471416
H	-15.84689219080660	4.68902954696465	-17.69812892912143
H	-16.90779586778249	5.43097948620662	-16.48045430069912
H	-16.22369897529956	3.81533829119242	-16.19549764099245
H	-19.27091891273123	4.46108960331919	-16.08119721038042
H	-18.55135666684311	2.85451618462283	-15.81447660700197
H	-19.81676355165584	3.07124569506322	-17.04983440530508
C	-24.30464022713743	5.78471160714330	-17.82764190938302
C	-23.73254179297200	5.22766439657638	-19.13167657047967
S	-22.20636209527954	5.98431910089058	-19.68269945354125
H	-23.60222847462073	4.13832010305713	-19.07263528036096
H	-24.42624210966997	5.43483468762472	-19.95986024722349
H	-21.41566847966076	5.59406522086354	-18.65456319592625
H	-24.34885029731687	6.88256918620003	-17.85871737855398
H	-23.68287531658902	5.49772808871460	-16.96668885229239
H	-25.31886720366684	5.40455644343688	-17.65807191029421
C	-27.47047789197384	3.77918450169040	-17.15704403479567
C	-27.54374444851832	2.28997223099401	-16.80396599073066
C	-27.23579573352239	1.40890113914811	-17.99061562313373
O	-27.31592788126561	1.93223262737835	-19.11812348867058
O	-26.92972618314475	0.20370425229632	-17.80145297282589
H	-26.85003721255244	2.04725855344982	-15.98249856042044
H	-28.54942460815697	2.02917406940253	-16.42983468478794
H	-27.57696626400055	4.41394763966014	-16.26268263929714
H	-28.25603660827866	4.05707023003873	-17.87176333647177
H	-26.50753927802533	4.00350672259724	-17.62695025585728
C	-19.25006529884612	0.96832889192978	-23.09357993975660
C	-18.92046528271536	2.43660611576564	-22.76735113278176
C	-17.57386446184568	2.59592676411678	-22.08052931065579
C	-20.03718912022929	3.03512423985171	-21.89609018590725
C	-16.99089352953315	3.99452896916261	-22.21720999363012
H	-18.88616354083625	2.99377570309605	-23.72178085887079
H	-17.67483285879525	2.32547565174254	-21.01435405841769
H	-16.85710643985628	1.87313216767769	-22.50883549114004
H	-19.82742973138087	4.07496469789323	-21.61523096362022
H	-21.00741577275807	3.01932227242160	-22.41529588440455
H	-20.14212054524498	2.45122220582908	-20.96734596995505
H	-16.03136656900769	4.08696503650496	-21.68676987141819
H	-16.81307319816401	4.24022505048564	-23.27581669881937
H	-17.66537358371770	4.76078884835394	-21.80965166009884
H	-19.28038085000504	0.36457019398482	-22.17175063842045
H	-18.49340585167535	0.52730579446586	-23.76040469429444
H	-20.23138525927636	0.88220564361811	-23.58361628470614
C	-24.01092325789259	9.24720702242573	-22.90830680359214
C	-25.06635417605449	9.48283968832397	-21.82606207406142
C	-25.36484967300905	8.26513340912421	-20.97616882872502

N	-25.84194204404926	8.51836982415513	-19.76375653904336
O	-25.23534010485696	7.11555036002074	-21.41375781025418
H	-24.78028864256731	10.32701519020419	-21.17954879849030
H	-26.02439837643619	9.77218788859422	-22.29243834977736
H	-26.15317094078438	7.75463257335240	-19.17067539911532
H	-25.94835674045506	9.46550133093556	-19.41652013826493
H	-23.89624377275151	10.14602412140143	-23.52941085775652
H	-24.29583646705605	8.41112271554104	-23.56038836845492
H	-23.03625326797513	9.01327318738334	-22.46300912008662
C	-20.40360113927920	8.11117649451431	-22.54919724496796
C	-19.87088500387428	6.68600901789365	-22.73264520095490
H	-19.13119422342414	6.63310850707094	-23.54509576879542
H	-19.38324458283217	6.32611535704045	-21.81563733492473
H	-20.68188008637271	5.98344694679147	-22.97072441020775
H	-20.90494434713644	8.47210993081323	-23.45864124039405
H	-19.59010683198997	8.81441534046124	-22.31573978236942
H	-21.12851438311460	8.15698718973030	-21.72410761867226

Optimized atomic coordinates in Å – oxidized *Ng*TAL Cys90 cluster model

C	-25.35750762060743	8.40612173155374	-7.91381001573761
C	-24.55437862211434	7.69929081657047	-8.99308146979463
C	-24.35557309809504	8.53337024425591	-10.25587855529982
C	-23.56452634753016	9.81680245357157	-10.01507700445887
C	-23.69628140460636	7.72030224185718	-11.35505516009228
H	-23.56510576481441	7.40652092911682	-8.59555034511686
H	-25.06188448325769	6.75971230341357	-9.27097173433471
H	-25.36273780551478	8.82505841338502	-10.61166593352652
H	-23.46329610392472	10.39219413459237	-10.94531088483773
H	-24.04319793011467	10.46987493721281	-9.27144868188852
H	-22.54890088172335	9.58314443400532	-9.65417147472574
H	-23.61201874596973	8.29882505176883	-12.28740939626623
H	-22.67796111579377	7.41555289057217	-11.06055431258519
H	-24.26576260084392	6.80433330175658	-11.57821388971291
H	-24.84250782836513	9.30252226935884	-7.53868944976069
H	-25.53813975772733	7.74765895240776	-7.05094913215647
H	-26.33912826543393	8.72511301328817	-8.29925909791338
C	-28.86698430467195	12.77726757199177	-10.54823876219482
C	-28.80314795690449	11.59941640354628	-11.52509528534509
C	-27.54999288414292	11.52747700594161	-12.38795626809708
C	-27.66249007039608	12.55721621982682	-13.51596003311780
C	-26.27836505609128	11.74618181792276	-11.58209683221887
H	-28.88813153371741	10.66882923436683	-10.93556697399135
H	-29.68860544421921	11.61819927667603	-12.18270573969012
H	-27.50745503985673	10.52409047431674	-12.84557799839127
H	-26.77724099920612	12.53266197269722	-14.16992896738822
H	-28.54818393718290	12.36545229755153	-14.14067661668961
H	-27.74829655307587	13.57801743731027	-13.11178630126316
H	-25.38871183522900	11.56657636999064	-12.20358759220123
H	-26.21573812451326	12.77743653091580	-11.20362237396230
H	-26.22306872078653	11.06723216964151	-10.71767993092380
H	-28.74816233781955	13.74578748772253	-11.05772939350869
H	-28.08563099188616	12.70782654765946	-9.77852164993003
H	-29.83797538659910	12.79617579389923	-10.03180359228547
C	-19.60397642860559	12.23804422405420	-13.89320253067740
C	-19.95675855702162	10.76178728635951	-13.60479274478339
C	-18.77661798258703	9.98912545926731	-13.03297870039990
C	-21.15764028171577	10.64985792253333	-12.68775080387211
H	-20.22425397089757	10.29970165095750	-14.57174502949841
H	-19.03586094153458	8.93134771561417	-12.87336292013927
H	-17.90320109282479	10.02554961608737	-13.70222152877478
H	-18.46988861187964	10.40766248723472	-12.05977365718230
H	-21.43453055862340	9.59939246051377	-12.51558985112315
H	-20.94438362566324	11.10111693569384	-11.70462682672773
H	-22.03860401707841	11.16312885349189	-13.10385464066351
H	-19.33039724363866	12.76071605681082	-12.96195683305410
H	-20.45986835203623	12.76623688548944	-14.33912269137457
H	-18.75360326841055	12.31519455879596	-14.58724690042781
C	-27.55609294395730	9.07901811096936	-15.94658772497160
C	-26.14187315886941	9.60586605064535	-16.24902020604662
S	-25.11338360912664	9.89084108189112	-14.78423715562382
H	-25.61312506476776	8.87923400618353	-16.87887554679779
H	-26.21431751820039	10.54667404910502	-16.81230528117832

H	-23.93520223243517	9.98495623105128	-15.44173377534170
H	-28.12541199273026	9.78410626191652	-15.32416655599591
H	-27.51931173941575	8.11097000699669	-15.42715065139708
H	-28.09395654136710	8.94658430608570	-16.89532841749228
C	-19.92524247584666	9.91661563746224	-17.96097574960357
C	-20.37916602595195	8.52539548892809	-17.47665382817751
C	-19.31328266248702	7.91087846515409	-16.57699910155295
C	-21.72230523187566	8.59104765197584	-16.76979083789797
H	-20.49180587664258	7.88043604216414	-18.36697663961768
H	-19.60275459409471	6.90420673875273	-16.23760452739041
H	-18.34716805658122	7.82704878464324	-17.09765387486220
H	-19.15715896937477	8.53242983297421	-15.68094766934139
H	-22.05712399781813	7.59357893725875	-16.44629402381070
H	-21.65942663727597	9.22636819956262	-15.87077613070231
H	-22.50259545302434	9.01301907198854	-17.42206540844780
H	-19.78647427915555	10.59424878277100	-17.10284439757554
H	-20.66977722647012	10.37066443849986	-18.63242452585833
H	-18.96890373278255	9.85524960056975	-18.50145576642793
C	-24.03694395658719	9.23160901687033	-22.90154391642363
C	-25.08736235372936	9.46907218454743	-21.81483175073964
C	-25.37757111399456	8.25446107980934	-20.95767047638654
N	-25.84902798123762	8.51192335453041	-19.74394440592068
O	-25.24680547990630	7.10323793462277	-21.39054877523998
H	-24.80177781872596	10.31779988735497	-21.17419951141147
H	-26.04724990627800	9.75323106981684	-22.28048658685279
H	-26.14086183182476	7.74798620151466	-19.14065958685077
H	-25.95945096037304	9.46092993452560	-19.40327178011555
H	-23.92111423474439	10.13176305216793	-23.52038161832280
H	-24.32925965630050	8.39944897311369	-23.55530138715503
H	-23.05803320078962	8.99099469000623	-22.46244390036117

Optimized atomic coordinates in Å – oxidized *Ng*TAL Met1 cluster model

C	-29.05666684863780	21.63963432862421	-1.84337391963651
C	-29.89709692872169	20.39470969265357	-1.59606789987325
C	-29.66821557132414	19.31949605397554	-2.64590289759621
S	-27.97062770251003	18.71234572414623	-2.71791588755317
C	-27.81275931893934	18.05563853790407	-1.06450579929976
H	-29.69549112579556	19.98808397342869	-0.59281653747035
H	-30.96976310577032	20.65312024497509	-1.60575017743628
H	-30.34605749978251	18.47003119266118	-2.49409832403213
H	-29.86600807688160	19.71660910851531	-3.65412936197158
H	-26.85499854631804	17.52143411305603	-1.01377553712801
H	-27.80867989287793	18.84609487321977	-0.30252700548336
H	-28.62200021061271	17.34254922543709	-0.85086185618324
H	-29.19440731464633	22.38676622395067	-1.04828684194729
H	-29.32893776055808	22.11183655028503	-2.80066075547498
H	-27.99025816099213	21.38654303562994	-1.89276557882236
C	-30.55295795206884	16.66251204899802	-6.48804734803589
C	-29.69359547928719	16.29561985204453	-5.26886391054745
C	-30.38210887741253	15.34605996100178	-4.24473065080204
C	-31.13296680077263	16.10027038627341	-3.16347604553084
N	-30.88843334451641	15.75533762714171	-1.89794488926069
O	-31.91483211498870	16.99289035388600	-3.46349106293416
H	-28.77855448303320	15.79439422031267	-5.62074575263850
H	-29.36028607163556	17.20437141669032	-4.74630992845019
H	-31.11217822057288	14.70397633228800	-4.76454852594417
H	-29.63594921118595	14.67550163346238	-3.79421701465361
H	-30.23463433341655	15.01855286027384	-1.65697353060528
H	-31.38729415650654	16.22005032822953	-1.14424031953555
H	-30.84995480135162	15.75886790693824	-7.04249421107889
H	-29.99526421040249	17.31434833742149	-7.17666796135203
H	-31.46825610895857	17.18989463458507	-6.18551850712216
C	-23.94781304577420	23.01910036939581	-1.17162822084700
C	-24.34292176332210	21.53183163249160	-1.09307377731546
C	-24.52126030027474	21.00646682011268	-2.51588102904893
C	-25.59191099081713	21.33228618498188	-0.25242259966420
C	-24.52897498831262	19.51685993350400	-2.60375496047852
H	-23.50663035178706	20.97881848658432	-0.62644225010278
H	-25.45981323298717	21.42260848847137	-2.92646471996422
H	-23.71098858469173	21.40835018701489	-3.14839318360730
H	-25.92005314274376	20.28327417676430	-0.23950535140550
H	-25.42334297009249	21.63804029460055	0.79185284353527
H	-26.42091855209429	21.93938567755830	-0.64516618043038
H	-24.70976080184777	19.16174749681036	-3.62983125102762
H	-23.56232561195034	19.09800996434430	-2.27802544057985
H	-25.30823289998489	19.07027389754298	-1.97220878147018
H	-23.81415581141134	23.46068943764130	-0.17169352843350
H	-24.73250569567783	23.59492568473610	-1.68909766467743
H	-23.00916002575172	23.14779248943508	-1.73055586608239

Optimized atomic coordinates in Å – oxidized *Ng*TAL Met32 cluster model

C	-19.13833078225849	14.83774007090101	-9.96947833594477
C	-19.89516806848588	13.68031111797444	-9.31788949230751
C	-19.16763017803022	13.02386416051211	-8.15528850888934
C	-19.11442294243511	13.88856252990519	-6.91851135817051
C	-19.80633834268707	11.67875199453830	-7.84617526998768
H	-20.88651775942644	14.02540446927498	-8.97148800465572
H	-20.09378837112450	12.91382217261836	-10.08632369671106
H	-18.12738015887533	12.83171017227027	-8.48358805755081
H	-18.54744770074949	13.39734723121170	-6.11307000494533
H	-18.64035140747199	14.86323078886849	-7.10927869001322
H	-20.13138182982360	14.08323272380251	-6.53694835124210
H	-19.31219258965722	11.17257239018047	-7.00313734231915
H	-20.86968340013521	11.80246855901484	-7.58387155940995
H	-19.76076174582433	11.00795260245278	-8.71591816625997
H	-18.94943219709431	15.65610756918444	-9.25831131902829
H	-19.70259486405946	15.25950893619729	-10.81429308911415
H	-18.16316030652584	14.49917027057275	-10.35325545708632
C	-21.14256993513178	5.08402495943063	-5.76486365213759
C	-20.27703552630249	6.08052869961616	-6.52287716540047
C	-20.98799903930426	7.29935920412021	-7.09693074088170
C	-21.42814722825512	8.27439181290427	-6.02587267257643
C	-20.08710361528610	8.01409149805311	-8.08632156666717
H	-19.45709321752568	6.43170542449749	-5.86898222617465
H	-19.78290690624379	5.54727909399735	-7.35414935761093
H	-21.88328618576184	6.94250611807046	-7.63883265279550
H	-21.96548553655501	9.12945540414236	-6.46351110665591
H	-22.09613243522888	7.81132965964127	-5.28371018766507
H	-20.55413909785350	8.67355498820162	-5.48499030005638
H	-20.59210724097119	8.88219357449298	-8.53375115425505
H	-19.17650627610946	8.38329927938044	-7.58667673860320
H	-19.77277364245060	7.34766929730265	-8.90470835706562
H	-21.62774268002558	5.54091739597356	-4.88858789327843
H	-20.54416280262945	4.23471908658385	-5.40414066975908
H	-21.93907822501150	4.68139032171475	-6.41056962061478
C	-25.36357972252711	8.39964392770830	-7.91244205232877
C	-24.56436824966875	7.69350561511886	-8.99506970556773
C	-24.36708005496042	8.52975178806693	-10.25667118594992
C	-23.57308802414899	9.81124097991794	-10.01521595907732
C	-23.71194928460493	7.71768126176356	-11.35906832670036
H	-23.57561690011551	7.40003726109708	-8.60125335201624
H	-25.07269320248525	6.75449689183486	-9.27346966411131
H	-25.37500179303083	8.82455537794032	-10.60884339429273
H	-23.46357530509847	10.38516433038294	-10.94763205062775
H	-24.04946349053203	10.46745971317464	-9.27299524718858
H	-22.56047551522454	9.57664030781100	-9.64648757617762
H	-23.61830208770760	8.30106821560255	-12.28773251845322
H	-22.69727629873986	7.40345206188348	-11.06152903265325
H	-24.28682879707588	6.80684045246195	-11.58866487237644
H	-24.84687878759490	9.29784900497868	-7.54192558482564
H	-25.53681348683048	7.74268962796600	-7.04702091533531
H	-26.34803035436859	8.71714144179987	-8.29151889219078
C	-27.42196842070608	12.79936326121007	-6.98899729267429
C	-25.92860488566851	12.55211861889303	-7.22578702928396

C	-25.08274789694067	12.32055398756051	-5.98702395549522
S	-23.36704947776854	12.18454443405998	-6.52022450652770
C	-22.58751920143409	11.70967117408534	-4.96355641489371
H	-25.81055960234911	11.68422917373442	-7.89543839226157
H	-25.51419979058130	13.41388865692808	-7.77750741996045
H	-25.18087871664297	13.15267534495692	-5.27191390882522
H	-25.37479978682097	11.39030890749466	-5.47466410092363
H	-21.51497435162318	11.58799475576074	-5.15794744973080
H	-22.72570398088097	12.48928543290046	-4.20228804821973
H	-22.99479087681203	10.75720703868739	-4.59968127787751
H	-27.94059645151879	12.94957756009477	-7.94609407528210
H	-27.58672210819936	13.69421083706531	-6.36961900762026
H	-27.89400986202338	11.94597897945363	-6.47841402471524

Optimized atomic coordinates in Å – oxidized *Ng*TAL Met78 cluster model

C	-9.97880679549700	0.31800167784734	-6.83612092968669
C	-9.03064761524768	-0.03949687776962	-7.97231235301208
C	-9.65010349794402	-0.11213674355501	-9.33608794529908
C	-10.94458614666848	0.32465032690234	-9.62123475093066
C	-8.88711928900445	-0.61563295481471	-10.37561026369869
C	-11.44213502368106	0.24289606628380	-10.90787661924410
C	-9.36868394053998	-0.68840775631934	-11.64522647062522
C	-10.64016231992189	-0.23363303453471	-11.92733226764980
H	-8.20490707358266	0.69324107588887	-8.00162580805504
H	-8.54747748554867	-1.00766248964488	-7.75872269909545
H	-11.57708302010139	0.71607821410659	-8.82306126448727
H	-7.87647147173874	-0.97496555399766	-10.15853061452781
H	-12.45865922631504	0.57988988417927	-11.12173519673585
H	-8.74613509862995	-1.09394324372172	-12.44556527181024
H	-11.02538144654234	-0.28169780814807	-12.94674478536292
H	-10.41510674803522	1.31926380364201	-6.96749964465434
H	-9.44627271543643	0.31401121778854	-5.87457774772154
H	-10.80885560113233	-0.40092752623945	-6.76278441252858
C	-10.65809760115847	-8.71689545893854	-9.09914466649114
C	-9.75582845001574	-7.89703735159837	-8.15944415042320
C	-8.30384468203740	-8.31854949740272	-8.30210341128928
C	-9.90884678531517	-6.41973272141560	-8.45643811934445
H	-10.07772564586553	-8.08777967136337	-7.11981304554356
H	-7.64926397876771	-7.74246631575608	-7.62993162827253
H	-8.16525760526560	-9.38665539634262	-8.07281393386799
H	-7.95147282086949	-8.14983604187657	-9.33267807022465
H	-9.27796796486351	-5.80330518554035	-7.79714509316282
H	-9.61690187486949	-6.20151236404437	-9.49560393656257
H	-10.95026395579819	-6.08665110540414	-8.33017512248766
H	-10.57832886766316	-9.79573506200033	-8.89590960342684
H	-10.36703255030284	-8.54784167868414	-10.14873844186255
H	-11.71422410426654	-8.42651791272286	-8.99178704680758
C	-13.66381191890645	-3.06254378170426	-14.15609540932576
C	-12.53696949617622	-2.97186037335837	-13.11971918861326
C	-12.07610159645942	-4.35543034348952	-12.65729413956974
S	-10.48507420240467	-4.37378648948867	-11.82146887171684
C	-10.92353635723628	-3.60084255877524	-10.26735510568430
H	-11.67783061812762	-2.44610896363212	-13.56182725968675
H	-12.85966915083886	-2.37323373025117	-12.25500546409763
H	-12.82918301828678	-4.83652687718008	-12.01313217594053
H	-11.93388488494117	-5.01056387033689	-13.53200652308488
H	-10.02762857559412	-3.61085658861112	-9.63473859725385
H	-11.23382285046257	-2.55860278210974	-10.41240278501213
H	-11.71861258129461	-4.16667283161651	-9.76318883303445
H	-14.54206015227322	-3.58811210055812	-13.74999439186761
H	-13.32861921640332	-3.61087776773285	-15.04959096527029
H	-13.99041967390272	-2.06174507570685	-14.47412297375759
C	-4.11204937640814	-6.65122681361791	-10.95992515783492
C	-4.81250738405816	-5.70813725556642	-11.94198048357934
C	-5.71882985472104	-4.67527868278740	-11.27720026067523
C	-6.37522167102901	-3.81713681197132	-12.34827276618176
C	-6.77013932373884	-5.28255099059745	-10.37190100935104
H	-4.05814602783918	-5.17769780842003	-12.54660189318946

H	-5.41502247772063	-6.30279026376192	-12.65295467196300
H	-5.07523223632108	-4.01976885836190	-10.65917490008964
H	-6.98658488861057	-3.01452391097611	-11.90790006472370
H	-5.62701561657279	-3.34903824797609	-13.00676916537976
H	-7.04050032836818	-4.42854546877012	-12.98000779883140
H	-7.42532337446218	-4.50309417625077	-9.95409219497295
H	-7.41213473482703	-5.98449192575224	-10.93005055553777
H	-6.33349036953205	-5.83374501538064	-9.52735674180886
H	-3.40720596951906	-7.31618011616271	-11.48084376300946
H	-4.83063226537909	-7.28726304878453	-10.42376004239682
H	-3.54108840495805	-6.08477898511406	-10.20689053166615

Optimized atomic coordinates in Å – oxidized *Ng*TAL Met136 cluster model

C	-22.47959158240357	12.26474903619669	-19.48652941121030
C	-21.61218593237864	13.19807256064263	-20.34187181958265
C	-22.20746278670356	13.47554660282563	-21.69742054061596
C	-22.07574907113132	12.54998496707777	-22.72708011958216
C	-22.93938281959209	14.63762646163149	-21.93927983933566
C	-22.65591079334173	12.77452006761392	-23.95461588993735
C	-23.51055563921416	14.85815447025902	-23.17582453217839
C	-23.37581960835040	13.92660420916005	-24.18148308185274
H	-21.46245398556732	14.14972638953514	-19.80866902763070
H	-20.61458495112501	12.74961525653954	-20.47143236092712
H	-21.51266269568787	11.63029453992494	-22.54909435668020
H	-23.04711758326699	15.37859583816991	-21.14325475006180
H	-22.54191422369844	12.03688767595132	-24.75226041345675
H	-24.07502614236537	15.77635305799146	-23.35531265138589
H	-23.83963866437748	14.09485863271504	-25.15437315255241
H	-22.00873901978860	12.07780935151726	-18.51030662949579
H	-22.62451499346964	11.29529131394865	-19.98607419036209
H	-23.47296550872091	12.70336873094112	-19.30886497240251
C	-17.60903525207325	10.50137806724714	-23.44849772107514
C	-17.47505359996891	11.90964303738007	-22.85634873647630
C	-18.52924681712503	12.87469979525101	-23.31878892254585
S	-18.45189112267856	14.41815179679296	-22.39065105395041
C	-19.51265339724952	15.44491111664180	-23.44011449521058
H	-17.51263174367694	11.83002607523045	-21.75534048429779
H	-16.47782883310159	12.31578409061310	-23.09367304266448
H	-18.39781510521256	13.11239297424712	-24.38702632184579
H	-19.53503197771590	12.44454749947541	-23.20064409465542
H	-19.58580693321186	16.43003046150055	-22.96453709394553
H	-19.07596169586747	15.55717424470996	-24.44119844717569
H	-20.51785997537084	15.01070271074077	-23.51280787611002
H	-18.57212852222731	10.04612853420320	-23.17226530688364
H	-16.80574210258794	9.84783044151886	-23.08058402758697
H	-17.55114190118850	10.52349195087511	-24.54772562710735
C	-21.02952828813975	16.59319167413315	-27.63818830636976
C	-21.90137593839492	16.31806053278660	-26.40617659980618
C	-23.36950962140313	16.61135925888863	-26.62285990763480
C	-23.71509404828746	18.09845618180564	-26.61927250565427
N	-24.94434282780848	18.40562589386486	-26.95320051570949
O	-22.90252733142449	18.95140009058909	-26.28951947691283
H	-21.79465950541562	15.26156166380039	-26.11682053186280
H	-21.53735209024039	16.90932144314733	-25.55218131742407
H	-23.73022182557185	16.15868522583192	-27.56209814260223
H	-23.97632146044945	16.15400263141530	-25.82450107067697
H	-25.62135804868427	17.70400655249531	-27.23576634114151
H	-25.24407342832607	19.37655036392673	-26.94299144242433
H	-21.09041092396539	17.64823272518724	-27.94193672596803
H	-21.35338874324665	15.97651187806096	-28.49111778428940
H	-19.97384194117893	16.36040288774587	-27.43634644695490
C	-16.26270085735531	19.41793280032935	-23.59238585921395
C	-16.75540275801603	19.02078761584733	-22.18169152063472
C	-18.24831235094892	19.12823299012388	-22.12025323796684
C	-16.28637872175011	17.61386998644114	-21.76644176273771
H	-16.32027208491629	19.74113977225219	-21.46484736824448

H	-18.64199936999089	18.85420874577335	-21.12848445270444
H	-18.59355410423368	20.15003703248387	-22.34390426813551
H	-18.71981209003713	18.45758140254047	-22.85730327782010
H	-16.62695575073003	17.37352792791482	-20.74802395556329
H	-16.69675876638609	16.84805716444814	-22.44413035309657
H	-15.18898418840230	17.53267474006219	-21.78826499505641
H	-16.58857617571120	20.43755261613877	-23.84639544430981
H	-16.67509210453950	18.73127055701780	-24.34958769976102
H	-15.16509867400450	19.38080568587715	-23.66336169853987

10. Optimized Atomic Coordinates for Reduced *Ng*TAL

Optimized atomic coordinates in Å – reduced *Ng*TAL Cys38 cluster model

C	-28.34354986053336	25.03139542461197	-13.67265787696412
C	-29.14085216597629	23.72718488686340	-13.52463183332644
C	-29.25772135814289	22.87365889588478	-14.79754196926260
C	-29.95226073944104	21.51409703303016	-14.54147051148387
C	-29.86137584042702	20.57901557100212	-15.76026034203007
N	-30.56873666408687	19.25657496764874	-15.59916637705377
H	-27.30943205319581	24.82832910510121	-13.99161345895251
H	-28.79904306423050	25.69635241621365	-14.42312960291339
H	-28.30165704709934	25.57869623216511	-12.71984662552515
H	-28.67082537215757	23.11110951202102	-12.73855261518660
H	-30.15522796863881	23.96000969941670	-13.15676901945768
H	-29.80355512519095	23.42992225157071	-15.57854566958445
H	-28.24557278086431	22.68503194241649	-15.19895586764652
H	-29.48429274420903	21.01949370912551	-13.67295280504445
H	-31.01114192443111	21.68106565750979	-14.27828939719150
H	-30.22709619348138	21.12994006180341	-16.64900586669386
H	-28.80209432587757	20.35773262963078	-15.95653941753502
H	-30.19217224511394	18.52989297355364	-16.22215867779292
H	-31.55198727266231	19.37745612274400	-15.85428538003822
C	-23.91865698224606	21.95598827702003	-15.08535040674092
C	-23.72618902011505	20.90053755033640	-16.17115660243188
C	-24.73325806282757	20.87297147597515	-17.23213491353781
C	-24.40893668974299	19.79311484329697	-18.22583916478754
N	-24.02381904327879	20.20130299040385	-19.42009609221415
O	-24.50209865795279	18.60505636568276	-17.92703939063792
H	-24.80689388779646	21.74404811634415	-14.47033438069151
H	-23.04448981297679	21.99708206067892	-14.42549131739011
H	-24.05019534166775	22.95761309578886	-15.52498485883270
H	-22.72946807260142	21.05336391129512	-16.62390338845619
H	-23.67170111495516	19.91218510872063	-15.68656051995882
H	-25.73501294121709	20.64986953008576	-16.82572673680524
H	-24.80543163840639	21.85167915894422	-17.73392603351149
H	-23.96603088021353	21.18565942791162	-19.65736701962983
H	-23.76525968774622	19.52210703087037	-20.13157306178449
C	-22.27194835013190	20.57406483489503	-11.96208267773096
C	-22.70645478101788	20.92477231049791	-10.52616435917171
C	-22.80197435418312	22.44380889563467	-10.31183353281935
C	-21.74594596211570	20.28550285032900	-9.52385288027187
C	-21.49101069303330	23.18978601630586	-10.47189573528006
H	-21.21538906077098	20.82324813370098	-12.13895353148764
H	-22.40384670140927	19.50129944586188	-12.15306373707249
H	-22.88003309569624	21.11572739670834	-12.69713348706947
H	-23.71423936157298	20.50570287740933	-10.36365280333329
H	-23.54378339374083	22.85165287740672	-11.02001836493704
H	-23.20797563251189	22.63043233054100	-9.30243675894204
H	-22.01798765754557	20.53457549124962	-8.48592387747745
H	-21.75016474660640	19.18858773468889	-9.61700762638714
H	-20.71239997009314	20.62291367752150	-9.69144259216899
H	-21.63673237534920	24.27169435293935	-10.33375335927989
H	-20.74002019918427	22.86759278625166	-9.73598193278603

H	-21.05725701556602	23.04591362185160	-11.47278404165783
C	-20.67775887864306	17.15066854238015	-12.47177759303643
C	-19.90425846647615	16.85400456545612	-13.76232455549455
C	-20.69469171346932	17.01110047958457	-15.02192641123314
C	-21.99156844916371	17.43434532958846	-15.12975731347365
C	-20.23295336475926	16.79423550566263	-16.35869369403082
C	-21.31043320007306	17.08665810848368	-17.22344011648924
C	-19.01642102052498	16.38380427843815	-16.91192838080087
N	-22.36521669029905	17.48048524576425	-16.44506775573495
C	-21.20535583030858	16.99569358044330	-18.59842703113398
C	-18.90831623637059	16.31081839026694	-18.27189460241704
C	-19.99779871232824	16.61224385349265	-19.10864393127873
H	-21.57152264994455	16.51492358142129	-12.38384246466761
H	-20.04497847468872	16.96857407036055	-11.59196569311332
H	-21.00276053764006	18.19673537256626	-12.43972576469536
H	-19.01435321927721	17.50756246591149	-13.80596156145574
H	-19.49837147709970	15.82736557014952	-13.71265292080062
H	-22.68967270000825	17.72568054931167	-14.35110337481002
H	-23.25472435585941	17.83705097725731	-16.81703782288113
H	-18.16591647085313	16.14445288672453	-16.26921267560630
H	-22.05191248074821	17.23277234240904	-19.24568053383565
H	-17.96446443883911	16.00370513770133	-18.72770155861667
H	-19.86928876536265	16.53457116411463	-20.19039914481446
C	-25.66930055889917	16.32622637494278	-14.03427174805389
C	-25.79533328039224	17.69418426059621	-14.72411191919515
S	-27.27159276065118	18.58734645266640	-14.27967242982147
H	-25.63484788413722	16.42908815489856	-12.94009807630339
H	-26.51085278123766	15.66677285553366	-14.29358140143976
H	-24.74030874632159	15.84328801019759	-14.36673243096601
H	-24.92500456717408	18.30938122637462	-14.45592164888780
H	-25.77081522457648	17.56118651207460	-15.81430441518572
H	-26.96586911780988	19.73226526855487	-14.92959076976640
C	-31.19620962766633	13.05662227253105	-17.24956174141463
C	-30.10857971566626	13.31000646850403	-16.20329531173386
C	-29.33223216963527	14.61131830895057	-16.40434286995204
C	-30.19131095838445	15.85830525546220	-16.29898889369958
O	-31.22608807907121	15.80816531669442	-15.60901399385857
O	-29.82324240811054	16.88798582425286	-16.90567805577113
H	-31.95041320930684	13.85603897017402	-17.23341001565766
H	-30.76895159326700	13.01550441068827	-18.26445865872288
H	-31.71006490919502	12.10153042892930	-17.06526810504239
H	-30.56624818664234	13.32309643941429	-15.20189471913427
H	-29.39584571339698	12.46780565271036	-16.20919711871362
H	-28.54119000544388	14.69764797694206	-15.63815884536958
H	-28.81148077533181	14.60934299516469	-17.37530219091739
C	-32.20670343728263	17.50677816741183	-22.25335948108043
C	-30.99517196623841	18.02512224804515	-21.48272685188831
C	-31.33890081712116	18.29557963447609	-20.01479674344435
O	-29.95045950159919	17.04566818078041	-21.56965501038088
H	-33.00000845358297	18.26825069560590	-22.28715797417482
H	-31.93044794823140	17.25359579929732	-23.28769915607633
H	-32.60672328974098	16.60315723479447	-21.76856572795293
H	-30.64380908967710	18.96565015124204	-21.95072213369669
H	-29.50000477218913	16.96134741387944	-20.68733943997347
H	-30.47005138419614	18.68302046592758	-19.46748286239238

H	-32.15016595213627	19.03486270736721	-19.93586510591972
H	-31.67078814666283	17.36601543843391	-19.52792304215449
C	-26.31791412487871	16.62043796089003	-20.04661437992225
C	-27.30631649838434	17.25290774781994	-19.05995780608276
C	-27.58744763377582	18.73387430842574	-19.35683862013213
O	-28.53404528796127	16.50796150521625	-19.14581506461944
H	-25.35504993530041	17.14776552039261	-20.01423812984943
H	-26.14988204581460	15.56373618962838	-19.79483890231459
H	-26.71835107624060	16.67710711473308	-21.07084551660990
H	-26.90260600254986	17.16016412769766	-18.03623200597556
H	-29.03821126584518	16.66080177625424	-18.27246184822769
H	-28.28629819460645	19.14816687529513	-18.61617133410904
H	-26.66696202703975	19.33056536068829	-19.33296433602319
H	-28.03696481384636	18.83607727248150	-20.35586976799765

Optimized atomic coordinates in Å – reduced *Ng*TAL Cys87 cluster model

C	-18.89556302608738	3.58286195157006	-16.37087338380524
C	-17.79779183784053	3.88741601886555	-17.40604449229521
C	-18.30364189861669	4.79802902026273	-18.50972808908182
C	-16.60714992183960	4.52241652854958	-16.71628998262200
H	-17.47782495528694	2.93168147582607	-17.85880674286273
H	-17.51531447021084	5.00994349742183	-19.24615624983450
H	-19.15476601615074	4.35653518429608	-19.04862141417300
H	-18.63822342823216	5.76254430413717	-18.09224577756640
H	-15.79100912540261	4.73200056029643	-17.42547155665105
H	-16.89640511920095	5.47974356912347	-16.25149819220941
H	-16.20250790091871	3.87663637704943	-15.92135768385052
H	-19.23221363638535	4.51386268517927	-15.88625784740032
H	-18.52441288158912	2.90771579336240	-15.58527348615092
H	-19.77276114155860	3.11158787716359	-16.84006248785391
C	-24.27267543474646	5.83012540377441	-17.76747458142741
C	-23.70598531946642	5.28877323077181	-19.07995506477905
S	-22.14406391827852	6.03130675122956	-19.59588966015372
H	-23.59312510192056	4.19676210388598	-19.04567220068897
H	-24.38440288946519	5.52935710960988	-19.91177377048776
H	-21.37854468465790	5.59163141050420	-18.56868533100290
H	-24.31302617106338	6.92851218045363	-17.78409115583601
H	-23.65061753799853	5.52790368427431	-16.91204916546171
H	-25.28797640003465	5.45242517284265	-17.60090266298687
C	-27.47516474052233	3.90698702036206	-17.15488201195321
C	-27.59618030140211	2.41896987797930	-16.87285586853426
C	-27.34239498118893	1.58781759642740	-18.10715784358016
O	-27.47180366884538	2.13383764696391	-19.22449887631618
O	-27.02216641735136	0.39565995288008	-17.96863673571357
H	-26.89400573006110	2.10792421046460	-16.08287390095568
H	-28.60309023685119	2.17345418401171	-16.49130135426122
H	-27.52628812579378	4.50697638430420	-16.23194068637923
H	-28.26924936532435	4.25304525864237	-17.82981817127825
H	-26.52022789270945	4.11746829873100	-17.64768794998452
C	-19.13930720420471	1.02804011504757	-22.85018273326916
C	-18.83529004219902	2.50520833341203	-22.54131704922519
C	-17.48753173343572	2.67986154924469	-21.83473996995585
C	-19.97364947173128	3.07349024866610	-21.68262157034162
C	-16.93338062969531	4.10548566770458	-21.96583989608544
H	-18.79798906054448	3.05799615349955	-23.49722705970375
H	-17.59541376286534	2.40580708973644	-20.77105129819854
H	-16.75450788088890	1.97502180603615	-22.26323078363377
H	-19.79980695097546	4.12070193332224	-21.40182994540240
H	-20.93756298392564	3.02737184411992	-22.21178846798686
H	-20.07070151173302	2.48941663173253	-20.75331019735363
H	-15.98316452093617	4.22131915302838	-21.42367304670727
H	-16.75077981458394	4.35179801875678	-23.02309440032641
H	-17.63478597190310	4.85086020132247	-21.56646523506566
H	-19.17006126118128	0.43937613641294	-21.91882860627778
H	-18.37052117878259	0.58606221036262	-23.50264343802315
H	-20.11456722497229	0.91931079286418	-23.34809913877078
C	-23.97290191707386	9.25256850116644	-22.87942220526300
C	-25.07472971028050	9.49402160293586	-21.84293881306295
C	-25.38913550433354	8.28689900767719	-20.99167582542232

N	-25.84781671202471	8.54948115424069	-19.77684245009916
O	-25.26199117937563	7.13848752145925	-21.41595513408779
H	-24.82304538769076	10.35126825447453	-21.19979990401888
H	-26.01493901733271	9.76613179229912	-22.35407376832831
H	-26.16633959217933	7.79131257893766	-19.18016521460670
H	-25.96347700010243	9.49937216542939	-19.44026875273487
H	-23.83930902313100	10.14377029902654	-23.50755369484421
H	-24.22524308572503	8.40549403288309	-23.53069506005633
H	-23.01587688530454	9.03199153273351	-22.39089718025948
C	-20.35086945371653	8.18848743869114	-22.45065500082132
C	-19.81649723770395	6.76626653585727	-22.61704358708253
H	-19.06507502873045	6.70780002386578	-23.41832363111867
H	-19.34155899685014	6.41063044691998	-21.69156139007392
H	-20.62337731079295	6.06110394623777	-22.86224315995884
H	-20.84662824945109	8.54213694537715	-23.36604690655363
H	-19.54040459515803	8.89542539257761	-22.21756166436561
H	-21.08119063147541	8.24131062072264	-21.63050144276980

Optimized atomic coordinates in Å – reduced *Ng*TAL Cys90 cluster model

C	-25.38673252154382	8.27880374058043	-7.85138875800302
C	-24.52996521718575	7.57947132387267	-8.89954125915116
C	-24.29257377222848	8.44193181405356	-10.13592679846661
C	-23.48836720909533	9.70059314349839	-9.83506560507253
C	-23.61262610756644	7.61327797409752	-11.22183500159755
H	-23.55569892164832	7.29089030908510	-8.46543883227548
H	-25.02200483864613	6.64212957046277	-9.20920907730590
H	-25.28384952068221	8.75289053488060	-10.51668222956023
H	-23.34709734787971	10.30223245443493	-10.74371516800604
H	-23.98031271939962	10.34040658465986	-9.08866486600934
H	-22.48903061865180	9.43917782921682	-9.44878023854971
H	-23.47383523386291	8.19282474733954	-12.14738408681227
H	-22.61679254665395	7.27704237003893	-10.88837812591859
H	-24.20288871401987	6.71769906574158	-11.46965909596038
H	-24.89318282548215	9.17532061971945	-7.44925851839709
H	-25.60556267239333	7.61712322331272	-6.99966598066609
H	-26.34988059363359	8.59488133537573	-8.28250118000760
C	-28.88404563334792	12.71045207279994	-10.56227423804829
C	-28.77354062524670	11.53114224355872	-11.52249630176019
C	-27.51870301602904	11.48768429772278	-12.38775780693022
C	-27.58948544582335	12.48383474179976	-13.52726785196654
C	-26.24980439935665	11.70182667939022	-11.57041584669172
H	-28.82235102758066	10.60121786501437	-10.92868148842524
H	-29.65812712792115	11.51045406028084	-12.18234869985336
H	-27.46649249976149	10.47694844389449	-12.83088430148110
H	-26.69497046874937	12.41891562263877	-14.16500439871767
H	-28.46979600308898	12.30432085301717	-14.16433662159936
H	-27.65481701335519	13.51843150267597	-13.15283345742306
H	-25.35641745202517	11.52806210155524	-12.18809252202227
H	-26.19084397058076	12.73094968380886	-11.18450645456166
H	-26.19978977597801	11.01739079126292	-10.70962476307523
H	-28.81178383182862	13.67733451785797	-11.08422745498528
H	-28.09523328828377	12.68727061065550	-9.79694041193834
H	-29.85122202828855	12.69329960879185	-10.03858967936452
C	-19.60422213448300	12.26329134981103	-13.80901149988534
C	-19.96094918175993	10.79022179599893	-13.50565990450438
C	-18.78568403744489	10.04587849544209	-12.91282443437145
C	-21.18057022445109	10.69379194622674	-12.60119308211766
H	-20.21738103224475	10.31472964882354	-14.46864967463749
H	-19.03068809457197	8.98799985029324	-12.73035493636315
H	-17.90762034301559	10.07692143638081	-13.57650471327725
H	-18.48804987973792	10.48819935803546	-11.94713395392981
H	-21.45980223833794	9.64578859808405	-12.41821880185345
H	-20.98047356673973	11.15985270438291	-11.62236449838839
H	-22.05310452728493	11.20153348563411	-13.04053393285401
H	-19.34108527751183	12.79616981858402	-12.88060896916343
H	-20.45297057969416	12.78825425316548	-14.27217051433353
H	-18.74492849777506	12.33005464661895	-14.49310743338393
C	-27.54213639907229	9.15233747746256	-15.85017575475375
C	-26.13399995725950	9.67392915460025	-16.18034280249778
S	-25.05601347529546	9.93470541392210	-14.76402184782023
H	-25.63495193841261	8.95321635075167	-16.84127480334981
H	-26.22166469789393	10.62028294122840	-16.73307792196901

H	-23.90067538056613	10.01178257931618	-15.46229917620820
H	-28.09298766646642	9.85131949048601	-15.20493776643270
H	-27.50012375747166	8.17649619067964	-15.34558610707451
H	-28.10271467609862	9.03662618576111	-16.78781718017333
C	-19.94284879418009	9.95458184592072	-17.85438982874283
C	-20.37864633202421	8.56168579459774	-17.36113930215745
C	-19.32654176730108	7.98165573806322	-16.44452223374446
C	-21.72759145007641	8.64574158713562	-16.66691571167123
H	-20.48180475311267	7.90387598274294	-18.24288022887959
H	-19.60974152672097	6.98126218839200	-16.08098569760286
H	-18.35357635988160	7.89009418694401	-16.95171120035601
H	-19.18003167087501	8.62447164028813	-15.56191328074888
H	-22.06905348796266	7.65835402179419	-16.31953467996088
H	-21.66820330192061	9.30392909040154	-15.78442003146347
H	-22.50152743120966	9.05286784405767	-17.33599341182452
H	-19.83031588638120	10.64191667395350	-17.00024543601080
H	-20.68556183319814	10.38819647527392	-18.54105853841640
H	-18.97711903983966	9.90845349532342	-18.38005705676651
C	-24.00109140290512	9.22684135856067	-22.87622034119486
C	-25.09818211383990	9.47080389897343	-21.83530916118761
C	-25.40416667305676	8.26766353303922	-20.97538397371318
N	-25.85724578341879	8.53585280804383	-19.75967356581247
O	-25.27556321133039	7.11721133293677	-21.39365237406116
H	-24.84684745318888	10.33316591932416	-21.19900055053581
H	-26.04046177453133	9.73661000071744	-22.34579156590361
H	-26.15127057817134	7.77838819692785	-19.14917034287427
H	-25.96693189686556	9.48743242579775	-19.42597378975211
H	-23.87024675520739	10.11727926129609	-23.50594158407856
H	-24.25839128028372	8.37983157639841	-23.52553833276784
H	-23.03841889110291	9.00521761430819	-22.39332894982330

Optimized atomic coordinates in Å – reduced *Ng*TAL Met1 cluster model

C	-29.08529171927183	21.55027541440235	-1.92509585129757
C	-29.92405206963575	20.30217591632693	-1.68245669479594
C	-29.73736251411853	19.24483327672246	-2.73573582746687
S	-28.04633084096892	18.67651873907408	-2.78254578319607
C	-27.89339498948477	17.99491801232121	-1.13438962986112
H	-29.70497277582827	19.88514435126703	-0.68756791589511
H	-30.99270401758497	20.58022596850247	-1.65341514527267
H	-30.40894480462742	18.38718109941707	-2.57460687914718
H	-29.94178389432538	19.64441112543553	-3.74146714276015
H	-26.94901897322154	17.43638818814150	-1.10220296392384
H	-27.86193407876598	18.77267128162308	-0.36040377325801
H	-28.71714138793122	17.29805637361287	-0.92393885157671
H	-29.24078587790723	22.29503158544382	-1.13130515691553
H	-29.35200240998108	22.02052570327563	-2.88500574905129
H	-28.01586244782768	21.30813191660334	-1.96088957395045
C	-31.19172689002839	16.34547088754966	-7.14766913312577
C	-31.01765874204886	15.77969566369452	-5.73819264758368
C	-32.24285832167231	14.98977707108137	-5.29064246575025
C	-32.17072914804946	14.51788273838730	-3.85549081919231
N	-33.31343971496737	14.53525337430196	-3.19146083666201
O	-31.10984753167631	14.13747817394145	-3.34946578792693
H	-30.12823208496406	15.13423041988632	-5.69130549132282
H	-30.83889219194153	16.60527574313621	-5.02965383265564
H	-33.15569474927180	15.58712659140267	-5.44488069026731
H	-32.35563717571821	14.09108466110340	-5.92296694769040
H	-34.17729144167149	14.85418171121812	-3.61686226115093
H	-33.35151197301165	14.18890917899133	-2.23629259523003
H	-31.35665350187779	15.54033693707978	-7.88060730561516
H	-30.30272878365604	16.91054501995808	-7.46335022925956
H	-32.05722015438106	17.02381028874643	-7.19746159559625
C	-23.97201584233649	22.93921410001006	-1.14806426797530
C	-24.34427225245394	21.44582851756319	-1.07188418165147
C	-24.49101247792842	20.89263717763893	-2.49028567965796
C	-25.58925897880769	21.23352469465910	-0.22028350254194
C	-24.52478078414685	19.36987478440792	-2.56538177092022
H	-23.50142321344415	20.91387930352287	-0.59139203173984
H	-25.41045262753155	21.31033203985869	-2.93965417456195
H	-23.65386132535378	21.26442327550334	-3.10475595721581
H	-25.88255434966465	20.17507389203784	-0.17231119738382
H	-25.42616426604932	21.58005231758884	0.81162539388818
H	-26.43962718338396	21.79901385168198	-0.63071995447958
H	-24.66581147000555	19.01953309565824	-3.59831372224096
H	-23.58149669233625	18.94042632811438	-2.19147032371238
H	-25.34220356353775	18.95194909032687	-1.96401125087902
H	-23.84256878212225	23.37565342824475	-0.14570360621945
H	-24.76607589592019	23.50689136627990	-1.66046330008339
H	-23.03671408855809	23.08513832425390	-1.70860089522729

Optimized atomic coordinates in Å – reduced *Ng*TAL Met32 cluster model

C	-19.13352324700398	14.86013509459156	-9.88037787301221
C	-19.90554619626992	13.73180801090829	-9.20046905206981
C	-19.15951228340219	13.06092347956804	-8.05647763947897
C	-19.02982878430832	13.94605383151961	-6.83168022756552
C	-19.83019787994708	11.71726290179500	-7.67926672233622
H	-20.87429787636247	14.10966887945713	-8.82563695674480
H	-20.15207447625752	12.96708528223168	-9.95690775088451
H	-18.14062733153392	12.82424845899333	-8.41880993614500
H	-18.44989970947607	13.44782368991300	-6.04010535969339
H	-18.52985231584110	14.89983241587889	-7.05775217006722
H	-20.02479399432725	14.18151569098499	-6.41747069206895
H	-19.29355561030913	11.21650630832374	-6.86058635147024
H	-20.86952847178796	11.88318588145249	-7.35331056862819
H	-19.85528866715642	11.03697725262477	-8.54198138342401
H	-18.90210802899341	15.67856647915422	-9.18214355023324
H	-19.70761647048864	15.29140000962522	-10.71372397004678
H	-18.17904018545477	14.49062228206344	-10.28709109642250
C	-21.22319486282839	5.02083710305325	-5.57900505632969
C	-20.34627430876905	6.04121222163503	-6.29436927615917
C	-21.03880902610176	7.30453687876925	-6.77009302986906
C	-21.43924017905628	8.18696868730889	-5.60226253216737
C	-20.12543946832499	8.05344661314647	-7.72215736044700
H	-19.50427919862968	6.33059145884879	-5.63841352679915
H	-19.88372100194589	5.54591544129704	-7.16648395680782
H	-21.95175854329244	7.01050493082983	-7.31949753120172
H	-21.97264062540654	9.08463391146628	-5.94881239265781
H	-22.09837613948194	7.66552389270121	-4.89270920002967
H	-20.54645959405353	8.52213901820071	-5.04882967835988
H	-20.60233414267454	8.96738097266639	-8.10415400924095
H	-19.19488945066661	8.35454287315244	-7.21344233886282
H	-19.84925661901240	7.43074660473995	-8.58703470635349
H	-21.67028448424776	5.43054592049941	-4.66045466542683
H	-20.64307877362465	4.13110280764192	-5.29337478718469
H	-22.04859932526907	4.68712832281279	-6.22776287327799
C	-25.39564521447310	8.27144527662507	-7.85631901587452
C	-24.54192692751047	7.57460179625918	-8.90860885176552
C	-24.30507255446175	8.44137882993439	-10.14207531061582
C	-23.49807734763673	9.69742047672524	-9.83775294339573
C	-23.62847173087555	7.61567942322405	-11.23231587536685
H	-23.56768452292270	7.28421644169957	-8.47892364179197
H	-25.03471322082967	6.63838038007576	-9.22064625993564
H	-25.29686153118905	8.75567633669516	-10.51976071933038
H	-23.33991963928440	10.29385896715901	-10.74916077932927
H	-23.99036027864994	10.34400709319003	-9.09765599380619
H	-22.50572323256978	9.43263196108119	-9.43621650338233
H	-23.47817169545072	8.20210719715736	-12.15196259326899
H	-22.63790992030406	7.26722741590295	-10.89533482261739
H	-24.22478395570151	6.72710011948651	-11.49038576774753
H	-24.90019312758505	9.16960148771113	-7.45780700982192
H	-25.60823413496476	7.61038801562160	-7.00264362803415
H	-26.36161275187800	8.58573591893173	-8.28208833940039
C	-27.60750059673542	12.71110758043991	-6.96571452159952
C	-26.11203460774184	12.50418365245763	-7.20612661162372

C	-25.29175546921230	12.19212217785032	-5.99545100953086
S	-23.55969063051694	12.08163168989769	-6.45610516757734
C	-22.85872978705289	11.61574630349971	-4.87254945003583
H	-25.97923361873955	11.69559069346034	-7.94548254786006
H	-25.70729647421879	13.41193961979812	-7.68782732289803
H	-25.40587208133103	12.97318575178205	-5.22538329580707
H	-25.59849483859729	11.23388141555111	-5.54449132013744
H	-21.77627641768168	11.50261595156491	-5.01050929011618
H	-23.04038666138461	12.39352537599296	-4.11778558538282
H	-23.27410246804800	10.66044803021209	-4.52467962858871
H	-28.12144562256447	12.93153601605912	-7.91159243852228
H	-27.78986584428870	13.55048013536075	-6.27717165266274
H	-28.07203082328741	11.81317486073559	-6.52982388070393

Optimized atomic coordinates in Å – reduced *Ng*TAL Met78 cluster model

C -9.97587826995552 0.33279944558128 -6.56592663200676
C -9.02858786217973 -0.04918955118268 -7.70385733737845
C -9.65588170385920 -0.12048566500517 -9.07567558183896
C -10.94838092453805 0.30810736908795 -9.34016702680019
C -8.89875783079626 -0.63143113453184 -10.12188978244426
C -11.45877927666701 0.23173849532055 -10.63083903112403
C -9.38617601087273 -0.69381569019943 -11.39252807687907
C -10.67168042707463 -0.27522571751174 -11.65601401299646
H -8.19232251770569 0.67046900162129 -7.73931883555145
H -8.56312397621470 -1.02440472823035 -7.48440247674101
H -11.57202723915987 0.70727318021386 -8.53925696380866
H -7.88318555094855 -0.98081137043652 -9.91286326870197
H -12.47826580866573 0.56451205930677 -10.83562885863646
H -8.76839854744009 -1.09782440910506 -12.19724179785168
H -11.07069186614457 -0.32955184912890 -12.66967435986480
H -10.39719858126516 1.33898012277651 -6.70677358255543
H -9.44102964581338 0.32902028412913 -5.60577473578859
H -10.81476905472617 -0.37478830820814 -6.48667905185847
C -10.58757616220232 -8.67421625505994 -8.79771160000798
C -9.68842910161449 -7.84187900438595 -7.86529577635865
C -8.24041346307507 -8.26239696034513 -8.00660524834484
C -9.83746425924106 -6.33872068922255 -8.16398222884088
H -10.01080578866553 -8.02227970186722 -6.82429044180202
H -7.58375789772216 -7.68646552931370 -7.33623372173443
H -8.10208195427877 -9.33035194295802 -7.77542889670485
H -7.88706761483658 -8.09715825071655 -9.03750495095007
H -9.19565371675728 -5.73478630242149 -7.50500768629441
H -9.55113898714407 -6.12581285634095 -9.20500582399121
H -10.87663458244074 -6.00500271316245 -8.02727758686141
H -10.50360603264288 -9.74936180051847 -8.57780442859806
H -10.29426029464798 -8.51966380614999 -9.84887203050600
H -11.64518144504151 -8.38663406142257 -8.69745674952535
C -13.70594177667539 -3.08928994825668 -13.90279319939854
C -12.59524064578233 -3.00707594785383 -12.84936030672196
C -12.08733639650427 -4.37842267983272 -12.40617589668851
S -10.50340194132169 -4.37132401696327 -11.56016984406332
C -10.94507449855580 -3.60367347107098 -10.00047052819364
H -11.74756560457320 -2.44304909998790 -13.26656255442803
H -12.94423215198281 -2.43712905829655 -11.97488875101586
H -12.83003953902354 -4.89808589565983 -11.77965637978233
H -11.91548165089030 -5.01392609738995 -13.29046596771614
H -10.04420121682687 -3.60432202195608 -9.37531132159209
H -11.26776186263018 -2.56515603565310 -10.14444785999975
H -11.73038717829449 -4.17931024089081 -9.49244957002762
H -14.57894581804312 -3.64557728053827 -13.52724401863399
H -13.34815089738014 -3.60018247500338 -14.80970503217751
H -14.04766746686943 -2.08465161824036 -14.19073013929490
C -4.08635476748980 -6.55412149771773 -10.68578376561373
C -4.80575346509820 -5.62751245553378 -11.66638470175769
C -5.70320404450918 -4.59038833297001 -10.99017245349543
C -6.32249838825172 -3.70078693920748 -12.06465924719176
C -6.77853444534502 -5.20525697225698 -10.12873698269953
H -4.06248389088886 -5.10008569542395 -12.28721592335479

H	-5.42022914094059	-6.22792930502050	-12.36162573573372
H	-5.05953274007936	-3.95927596728518	-10.34814008568118
H	-6.94110990495338	-2.90328430562565	-11.62502968313460
H	-5.55035413400662	-3.22533308846732	-12.68885182136644
H	-6.97243602590262	-4.29434177753857	-12.72839232805645
H	-7.43235487121495	-4.43080646496439	-9.69922289403837
H	-7.41827542037228	-5.88051715299247	-10.72134896170758
H	-6.36879565498118	-5.78890294400126	-9.29236569225820
H	-3.39226954883911	-7.22904549982934	-11.20855817375421
H	-4.79322782533637	-7.18088416505562	-10.12343220589662
H	-3.49995069209884	-5.97499320912816	-9.95465939117762

Optimized atomic coordinates in Å – reduced *Ng*TAL Met136 cluster model

C	-22.49826527518196	12.28095151861681	-19.42255632825016
C	-21.63072207212733	13.20251587899163	-20.29347503625285
C	-22.21752501294432	13.45997637295429	-21.65423164803222
C	-22.03621829613148	12.55451738891980	-22.69595391262275
C	-22.99557994359233	14.58493592277606	-21.88124273393095
C	-22.59495309356508	12.79002460573824	-23.94271804721663
C	-23.57430767677655	14.81244930141219	-23.11699136065088
C	-23.37499472452037	13.91799282912949	-24.14973021433932
H	-21.48581241957987	14.16281018853292	-19.77460422611142
H	-20.63126598914629	12.75379338970790	-20.40868345107378
H	-21.43814561204783	11.65553761179765	-22.52774073728882
H	-23.14547972347863	15.30265317951588	-21.07041312380842
H	-22.43080275671996	12.08086259436855	-24.75656465652581
H	-24.18441214894657	15.70456732553646	-23.27567927675382
H	-23.82944998268574	14.09012921030053	-25.12631029677578
H	-22.02788167597866	12.11268355704873	-18.44285114926770
H	-22.63999310784732	11.30276502832886	-19.90576494791135
H	-23.49278600478908	12.72053256670650	-19.25411500467838
C	-17.54771935490812	10.56293708334986	-23.28880905592323
C	-17.40826970711273	11.97517365319805	-22.71249717407910
C	-18.45449249054042	12.94204113727541	-23.20028097377623
S	-18.37082207736839	14.48443237735506	-22.27199003148796
C	-19.41038369829165	15.49405678303632	-23.32679529896063
H	-17.45936349796389	11.91063379797029	-21.61118728375162
H	-16.40685843128690	12.37328570284486	-22.94484259587656
H	-18.30521190980655	13.17465433035961	-24.26706205111859
H	-19.46444291849449	12.51978689759433	-23.09183126366180
H	-19.48735861628162	16.48734120550812	-22.86777421805815
H	-18.97182506677173	15.59731515501755	-24.32863954073589
H	-20.41876399832892	15.06595770034051	-23.40581519867480
H	-18.51649522450224	10.11816795587055	-23.01530422117136
H	-16.75284273178409	9.90640669146397	-22.90781813295624
H	-17.48115083190457	10.57183751462112	-24.38778043105005
C	-20.94282292260732	16.65637705606603	-27.51336949432447
C	-21.81309317053579	16.43096987189515	-26.27574685688669
C	-23.29322304638859	16.65097561753380	-26.51394835690657
C	-23.67912889253715	18.12699323894023	-26.53634907311172
N	-24.93928941087672	18.38795768916953	-26.83369875951698
O	-22.86091072000477	19.01704368868607	-26.28422227333231
H	-21.66751619701530	15.39956270443660	-25.91834450394642
H	-21.48040777268969	17.08932185203815	-25.45936725260623
H	-23.62420595504715	16.17644830785158	-27.45294173766021
H	-23.88936891491657	16.18146041413702	-25.71573515937205
H	-25.60813518340996	17.65104137024406	-27.03298901171068
H	-25.27078006116286	19.34841217392947	-26.84489110079789
H	-21.03914579045612	17.68584973148862	-27.88785150634860
H	-21.23417472079072	15.97303596338679	-28.32638706284802
H	-19.88109433176212	16.47743619541590	-27.28902377257045
C	-16.17216147512698	19.48077167687918	-23.44345288429023
C	-16.61532803597521	19.10341551187903	-22.01402476174347
C	-18.12824850963530	19.21457640139307	-21.89515986201485
C	-16.11120163859243	17.71654110957347	-21.63673435514606
H	-16.16187830278697	19.83350876850421	-21.31907702286014

H	-18.47779347505247	18.94942227683913	-20.88517802774754
H	-18.47295965550016	20.23656822876218	-22.11456758955173
H	-18.62465491461690	18.53684410004212	-22.60823835332792
H	-16.39867192369283	17.45058408984505	-20.60763980717752
H	-16.53393952134336	16.94861747364635	-22.30536253820428
H	-15.01441142020512	17.65603511089010	-21.70758853782704
H	-16.50624794904366	20.49502349492534	-23.70905552237269
H	-16.60549035831323	18.77924283937354	-24.17493057114357
H	-15.07712065850664	19.44323658603880	-23.54706362187789

11. Optimized Atomic Coordinates for Cys38Ser *Ng*TAL

Optimized atomic coordinates in Å – Cys38Ser *Ng*TAL mutant Cys87 cluster model

C	1.27192288817258	-13.21526066755980	3.85244460726854
C	0.75928685671789	-14.31252871596194	4.80419464165233
C	1.92130589205983	-15.03551906019408	5.52643746810131
C	-0.22940952288336	-13.72006845796656	5.80757607639793
H	0.22472319431158	-15.06524833083813	4.19734804345023
H	2.61924804817322	-15.48837390910059	4.80914085708798
H	2.49016040636060	-14.32367921617397	6.14634391949092
H	1.54131706481662	-15.82947703237237	6.18432360148773
H	-1.07435983742707	-13.23284011061317	5.29725223551484
H	-0.63780905880891	-14.49357573852803	6.47608661263443
H	0.26501572767279	-12.96124909035225	6.43639261368397
H	1.80609451746835	-12.43391251234015	4.41738922395011
H	0.43868971015726	-12.73530670497648	3.31742934445206
H	1.96788009384846	-13.62490376031086	3.10468238961510
C	7.24508440290820	-13.10323901995358	3.40761527800493
C	6.79079721999719	-14.56117477646520	3.41035101313499
S	5.88776793545676	-15.09608232343975	4.87779267314525
H	6.15918981759208	-14.74801936402072	2.53037042733058
H	7.67793201730655	-15.20477754012915	3.33288251858212
H	5.73325929833139	-16.38525391953747	4.50184133866876
H	7.79823574784852	-12.85294017170925	4.32565840616731
H	6.39812367766756	-12.40683210093030	3.32301934907504
H	7.91569225337931	-12.93763516897307	2.55985592947765
C	8.94089140140180	-12.62671939065279	0.04998014732305
C	8.27303103665457	-12.77836093922577	-1.30909172945003
C	7.83753234306465	-14.21498156187402	-1.57046517153151
O	8.28829546998759	-15.11049178818617	-0.81727193771400
O	7.04678949505079	-14.45029208396823	-2.50548587216686
H	7.39103702836412	-12.12317033313048	-1.39438046636750
H	8.95415741982803	-12.47003186197517	-2.12132832538415
H	9.86582485077316	-13.21533456108085	0.10728861282435
H	8.27255867410097	-12.99508773138071	0.83335182330596
H	9.18602902509818	-11.57537591261781	0.27211162081582
C	1.54793476661735	-20.17806237081350	2.92893921474336
C	1.90392916869625	-19.53740189313927	4.27993879908452
C	0.67058334458992	-18.91541381967135	4.93534225813083
C	2.98646942849150	-18.47560696126999	4.08345411708170
C	0.83149685269688	-18.67721270835708	6.43512189732793
H	2.29166464387910	-20.32732286625861	4.94860348372776
H	0.43257046640195	-17.96737598962792	4.42124035727916
H	-0.19925269273995	-19.57594997929873	4.77545498944444
H	3.92025109484295	-18.91428050630340	3.69948205371624
H	2.64535685726566	-17.72207913814931	3.35508349151584
H	3.22438320928536	-17.95136646771275	5.01677074285880
H	1.69714241588595	-18.03658006125208	6.65719209142707
H	-0.05694709314190	-18.19064592469447	6.86427317699427
H	0.98139018820251	-19.63023735048850	6.96598749431006
H	0.80172651989097	-20.97822732172694	3.04917251706212
H	2.43669572024523	-20.60943738431918	2.44365687777555
H	1.12620091939676	-19.42285556032881	2.24567275596445

C	9.65378129924083	-17.30755855554816	7.19885514509129
C	10.49836885054602	-16.12975308333974	6.70156290214360
C	10.03743181490665	-15.55709296315287	5.37353637513104
N	10.30792315025809	-14.27498376193539	5.18733100490642
O	9.51015090564488	-16.25279695451803	4.50697489087002
H	10.53731686061697	-15.33261539070962	7.45953006467702
H	11.54196432570624	-16.45550092341359	6.54834932813145
H	10.09987266628941	-13.83638769937623	4.29466269861251
H	10.73368936535865	-13.70358028306875	5.90922503175683
H	10.08173736267709	-17.72034826253974	8.12244187504462
H	9.61535697962206	-18.10802053798983	6.44808112116438
H	8.62480552144016	-16.99103115577890	7.40988540311175
C	5.96753549979420	-17.57847934537628	8.10377718551880
C	4.89776215698668	-18.12878311771463	7.16407002590234
H	4.09820207591128	-17.39227659832268	6.99823149636800
H	5.32174963952522	-18.38231717384485	6.18202529508292
H	4.42961683030611	-19.03860685598291	7.56801791574813
H	6.77149869332956	-18.30967354884853	8.27072984788316
H	5.54492916188268	-17.32011506940752	9.08648177273920
H	6.42441593399776	-16.66727755917514	7.69070703164158

Optimized atomic coordinates in Å – Cys38Ser *Ng*TAL mutant Cys90 cluster model

C	6.99266529031263	-2.60704474868714	3.73793197849176
C	6.32613890868513	-3.97456140111726	3.60778669115428
C	6.95739892339800	-5.05118793329672	4.50446339219710
C	6.90978616694543	-4.68444695412298	5.98938665058630
C	6.29216667516792	-6.38668716542283	4.26432624101222
H	5.25144044473516	-3.89335464716492	3.85186262367735
H	6.38138529829044	-4.31506419670368	2.56020143558127
H	8.02214127973072	-5.13546120533505	4.21375171870509
H	7.45039755378596	-3.75214677229006	6.20448074817273
H	5.86703177299681	-4.55699075993494	6.32433581234843
H	7.36090889135404	-5.47879050992092	6.60039916902878
H	6.77046311893750	-7.18953119667965	4.84615212154466
H	5.22982475575160	-6.35372110708960	4.55881659903580
H	6.33302464116518	-6.67346878418404	3.20166523618490
H	6.85547158141814	-2.17856961273831	4.74084239142507
H	6.57797090031843	-1.88887076830344	3.01480272551681
H	8.07601596838469	-2.67833949958859	3.55146508900821
C	12.86294228338519	-3.01569022748191	6.17195011395090
C	12.74759293031587	-4.23264652094178	5.25242691563118
C	12.00717603484720	-5.47631410561030	5.73808807486651
C	10.78459674661110	-5.24241841844537	6.55857189546674
C	11.68531547348875	-6.35239717037100	4.55601478033811
H	12.24692478613412	-3.88980692669725	4.32608602666030
H	13.75602100423826	-4.54427335692104	4.93274543435957
H	12.71662102119638	-6.03596686330543	6.38123486979584
H	10.99093695291081	-4.65684682215740	7.46740401859456
H	10.01009734455492	-4.69907368641674	5.98951888958479
H	10.33652239158667	-6.19438673096897	6.87874357950742
H	12.58118184105703	-6.58216927103042	3.95762109294210
H	11.23451521404425	-7.30387008775067	4.86533214673169
H	10.96324478209347	-5.85027342150016	3.88936844517521
H	13.36928368379574	-3.26055489794148	7.11981524226049
H	11.87528855609143	-2.60134444596428	6.42234942605675
H	13.44352611238037	-2.22162777701958	5.68088451360495
C	5.56355735489817	-8.34920338391671	10.51693758329151
C	5.13490624413608	-8.40332746263199	9.03567669772324
C	3.64106956455700	-8.10350662933017	8.89437764443605
C	5.95877589960319	-7.49153227331875	8.14220631203770
H	5.29703991712117	-9.44087370405837	8.69428768849255
H	3.41679107251434	-7.07910607234508	9.23583726889240
H	3.31436212827491	-8.18513179378378	7.84629087161155
H	3.03353143678682	-8.79761208895322	9.49472862067239
H	7.03313498911605	-7.72498864028110	8.20253554475048
H	5.65409344468890	-7.58647342546924	7.08998898467581
H	5.83023737286519	-6.43569984339988	8.43088671415819
H	4.96960843523467	-9.05009153380889	11.12187654672014
H	5.41754981970361	-7.33725021547166	10.92915900758829
H	6.62615817110651	-8.61108009912545	10.63351088259332
C	11.16527622576569	-10.12476851079654	4.23493131856733
C	10.28402365320732	-10.46965706820936	5.44732181645753
S	9.22520852996950	-9.13959649849364	6.04644650502714
H	9.57567259906480	-11.26800353830428	5.18197327315306
H	10.88883281691881	-10.84712010568133	6.28236189048280

H	10.18500432027328	-8.38740931653157	6.63176961338441
H	11.92763602905168	-9.38009907093285	4.49759073743543
H	10.56032265728338	-9.72675285613312	3.40880867755801
H	11.68018070929626	-11.03252552259951	3.88472553691410
C	5.46829487940315	-12.76706704176731	9.07598694288957
C	5.10247160260894	-12.58913985353844	7.58994378597601
C	3.72632862892395	-11.97022693543999	7.46603525127964
C	6.16799175785445	-11.77917265028085	6.84516132239693
H	5.06797323603684	-13.59536998084595	7.13355291339187
H	3.70392339315501	-10.96804548512560	7.92370354501746
H	3.42338537747964	-11.85865055046116	6.41297022072811
H	2.96406853603487	-12.58261958043201	7.97170978238810
H	7.15603846690363	-12.25808054207691	6.91727534080018
H	5.91768987015550	-11.67596541465180	5.77831243381527
H	6.25746761977255	-10.76550354177523	7.26675565128699
H	6.44608912833332	-13.25838492071160	9.19397291244790
H	4.71449390873139	-13.37480989190207	9.59871450816451
H	5.52051247974906	-11.78763079127979	9.57881969702795
C	9.68256985452108	-17.32839713175769	7.15254392450375
C	10.52404334718197	-16.14464705257810	6.66416756373984
C	10.05741020345734	-15.55882544436029	5.34389351630959
N	10.32588624610995	-14.27458343415335	5.16986744529286
O	9.52749445407472	-16.24612181139519	4.47224507573241
H	10.56679000764178	-15.35553515538902	7.43019606696139
H	11.56646631349579	-16.47064481727218	6.50409279118022
H	10.09842311155514	-13.82269306867916	4.28827932337609
H	10.75079488884566	-13.70945162896237	5.89717296691777
H	10.11466634749599	-17.74873639648342	8.07080339803005
H	9.64510498260055	-18.12197339419649	6.39446488047301
H	8.65062863632708	-17.01801783779963	7.37130291404788

Optimized atomic coordinates in Å – Cys38Ser *Ng*TAL mutant Met32 cluster model

C	5.56697145787903	-3.93187348149783	12.13023236316853
C	5.51023553706721	-3.54173678235228	10.65282301375612
C	4.28176001070683	-2.69325246829059	10.30409066776672
C	4.42367319127846	-1.26857050879530	10.81363745017188
C	4.01306693753081	-2.69405535282540	8.80756656525786
H	6.42318859080453	-2.99205057164124	10.36360039166448
H	5.49644547650827	-4.45991894501302	10.04109395686788
H	3.40949075433178	-3.15566163766829	10.80411916256002
H	5.26926275339389	-0.76741376397603	10.31432658086296
H	3.51773109863696	-0.67816292347988	10.60716901466275
H	4.60383222065054	-1.22894960735896	11.89843687434332
H	4.88118910659138	-2.31498394132593	8.24747525159644
H	3.81090066529129	-3.71163155645377	8.44513876286209
H	3.14697727646473	-2.06686778482142	8.54904060998078
H	6.45233943295376	-4.54633396863352	12.35203142819235
H	4.67582962449116	-4.51387760860527	12.41227016565319
H	5.60812141324361	-3.04811100148164	12.78453000149320
C	1.35738274552834	-2.18821983643264	2.60393962611916
C	1.28645183141474	-2.87458615003201	3.96420287075919
C	2.62311142326898	-3.00906529373877	4.68964446048227
C	3.12958714701696	-1.65811945454422	5.15444607092737
C	2.47045401347422	-3.97579006719061	5.85569928641156
H	3.34882067549206	-3.43708729335780	3.97540224918009
H	3.25528095318082	-0.95276675837333	4.32003892182727
H	2.42298137871640	-1.20791818872492	5.87097190641204
H	4.10336423920738	-1.74376976142828	5.66110639975027
H	3.42907447538212	-4.14850229502583	6.36484705608023
H	1.76403779050884	-3.57865815443296	6.60223603339441
H	2.08925360232047	-4.95057690689276	5.51461268509919
H	0.36938431767018	-2.15620105247195	2.12132075026049
H	2.04159196232529	-2.72627667544701	1.92891291078424
H	1.71754352310097	-1.15135036393495	2.68238146559027
H	0.85933447610058	-3.88294581555624	3.82514444451957
H	0.58014380075623	-2.33542906466249	4.62150029206155
C	6.98641077169046	-2.61230466864824	3.73734051630271
C	6.31147433608734	-3.97584476848066	3.60882505960329
C	6.94077440453853	-5.05707004031972	4.50133626306993
C	6.90223380869839	-4.69173910655352	5.98687011763031
C	6.26679847479166	-6.38847653288972	4.26285942426722
H	8.00338692517392	-5.14760297794835	4.20469575959545
H	7.46182620304080	-3.77116045091633	6.20478512915949
H	5.86279121776972	-4.53715655563945	6.32142995633664
H	7.33380179228074	-5.49795578547399	6.59918176569724
H	6.73538533843642	-7.19380573852080	4.84973335364499
H	5.20341496208256	-6.34480804616676	4.55276697685292
H	6.30815281518278	-6.67911059089445	3.20141558099971
H	6.35722896611139	-4.31597681979874	2.56061531671578
H	5.24153291957272	-3.89008682769670	3.86100010908090
H	6.86428919444014	-2.18797241152007	4.74473332300248
H	6.56445947520464	-1.88817675273837	3.02430002642351
H	8.06684301402684	-2.68698680021333	3.53527593706502
C	10.50960100676294	-0.06782990661459	6.32625995290269
C	9.23609939509421	-0.60739431011658	6.97585744240711

C	8.09087854912982	0.34616686778590	7.03810982591623
S	6.63820916745704	-0.58635769764079	7.55653106002551
C	5.39533304459839	0.69587489578857	7.46937289560408
H	8.91992944579573	-1.50580293614886	6.41678055764816
H	9.47072804496573	-0.96195155500519	7.99410037796054
H	8.27527707658121	1.16900725480368	7.74689226668397
H	7.88724730242357	0.79013282027034	6.04956367804802
H	4.43528190819591	0.24587523950502	7.75172948019541
H	5.61850314593539	1.51577181865023	8.16592545962850
H	5.31199899784535	1.09226198874525	6.44803638019043
H	10.89429753023997	0.81371994936180	6.86235766857155
H	10.32639153354706	0.22935896583141	5.28232381354819
H	11.29860532900905	-0.83288348432947	6.32700483470122

Optimized atomic coordinates in Å – Cys38Ser *Ng*TAL mutant Met78 cluster model

C -10.12937650373219 -5.74279443413578 4.04538877345391
C -10.83808520434246 -7.04054271831675 4.43053077761403
C -10.04479417906485 -8.30004167751356 4.17031410269813
C -8.67858706227718 -8.28429654948612 3.91336234618563
C -10.69168928739293 -9.52105134150722 4.19971004192838
C -7.97236005544519 -9.48551639269189 3.70774788990685
C -10.00837653278720 -10.70020140371830 3.98998926411095
C -8.64317519865314 -10.68345277447408 3.73503285505902
H -11.10577061740756 -7.00827730383783 5.50114848349300
H -11.79632569340000 -7.11604859359370 3.89139773256131
H -8.13467198138369 -7.33996086927474 3.87778684087386
H -11.76806950642177 -9.55030038622711 4.39242014597180
H -6.89899538899698 -9.45149137798611 3.51130241493667
H -10.54535498908565 -11.65067991987089 4.01458994528372
H -8.10825413336257 -11.62035276615380 3.56834551572520
H -9.22207269570602 -5.57842737545965 4.64443040926461
H -10.79192988587641 -4.88112864623616 4.20941810903847
H -9.83654159765086 -5.74587281405507 2.98479974429783
C -13.36781673865059 -10.38543668244602 -3.82604208836872
C -13.80826648666587 -9.22289360263399 -2.91923471736709
C -15.23727710520662 -9.45039419090035 -2.40251318872289
C -12.85189608090490 -9.05360076083410 -1.75402160833515
H -13.80267677548139 -8.29740537199205 -3.52248102718277
H -15.95461789893645 -9.54515434197202 -3.23189107970867
H -15.28539215974150 -10.37557825233909 -1.80599507464706
H -15.56678625518056 -8.61959159337114 -1.76022075497794
H -11.81412042348196 -8.90914858144990 -2.09006458749026
H -13.12901952286529 -8.19435782439638 -1.12384566091021
H -12.87849520573718 -9.95147672393326 -1.11988174746734
H -14.05266251897945 -10.51072905329416 -4.67821087492074
H -13.35753967233530 -11.33017317922803 -3.25812123671697
H -12.35380216761614 -10.22107022030176 -4.22083275926042
C -6.71989661104998 -13.36508613310130 0.23809719826964
C -7.82093201152718 -12.30968163509387 0.29799199466377
C -8.69574233923681 -12.38323696633168 -0.92688086243252
S -9.88086346307344 -11.04862041507085 -1.10542098527676
C -10.90118078459568 -11.34419252010295 0.33713530746974
H -8.42339781599182 -12.45317007397558 1.20732278741100
H -7.37619515312534 -11.30401217314867 0.37245477715321
H -8.07864253237497 -12.32500392206613 -1.83838403701506
H -9.23222832139318 -13.34580544982178 -0.96815340564775
H -11.40752755353476 -12.31754899988180 0.27275556713365
H -10.31211088172401 -11.29568282183168 1.26136192779112
H -11.65812518014316 -10.55388569650550 0.37790124829741
H -6.07608946697554 -13.21370358883172 -0.64191252913867
H -7.14429374122949 -14.37879307605759 0.17043504175583
H -6.08120013569133 -13.33010968162088 1.13281144968310
C -17.46832582590129 -12.03267669218220 1.90119727530028
C -16.18063928827377 -12.62552738372213 2.46703635265618
C -15.05960556309697 -11.61292170888041 2.71478897995022
C -13.87155635864352 -12.31545277679012 3.34821077812004
C -14.64772464492734 -10.91044493482958 1.42545059382483
H -16.40568939114321 -13.13962190589785 3.41728061711496

H	-15.79988735118310	-13.40488485768553	1.78185015743279
H	-15.43771119332166	-10.85108160481482	3.42206246535240
H	-13.05583620789878	-11.61115391784884	3.57096970560826
H	-14.15155975293952	-12.81528513513239	4.28840548703433
H	-13.46779876251817	-13.08392162621710	2.66838986411835
H	-14.30560583042284	-11.64447633792783	0.67740988518355
H	-15.47333477479454	-10.33842578245109	0.97982338866863
H	-13.81838080666508	-10.20926400742125	1.60397347849249
H	-18.25515876415683	-12.79740353895447	1.82128592026170
H	-17.31960518106147	-11.60836532569525	0.89779509586089
H	-17.85135578661399	-11.22811158647485	2.54919548857515

Optimized atomic coordinates in Å – Cys38Ser *Ng*TAL mutant Met136 cluster model

C 9.09499620902833 -13.53799510083712 10.00377403426743
C 9.00093976559656 -14.27341855784412 11.34012867271166
C 9.97807736177665 -15.39492510873774 11.45515117744678
C 9.70315740695247 -16.61323129481736 10.86239988591778
C 11.18230977655439 -15.21618808496870 12.10668422008572
C 10.59440214280066 -17.66086269963653 10.93597512824307
C 12.09450175009680 -16.25778857096981 12.18837367821548
C 11.79857993283838 -17.48512805389399 11.59850736402711
H 9.16274662736296 -13.55894544048924 12.16250248958732
H 7.97897538889791 -14.66546119649385 11.46651468004994
H 8.75114357277055 -16.74691446460129 10.34137957625341
H 11.40605046772624 -14.25420969404078 12.57458543997292
H 10.35655471743435 -18.62126709966831 10.47436332603164
H 13.04227285999433 -16.11795345405090 12.71229538122135
H 12.51460153104698 -18.30646159230291 11.65779186420503
H 8.35772733398887 -12.72353699269033 9.94827346989483
H 8.90768533224594 -14.22276428507912 9.16299835840732
H 10.09560434202809 -13.10176833052915 9.86297968748336
C 4.84623873297289 -18.10594126432342 11.63493401049622
C 5.26167660461385 -17.16652807476465 12.76938987937825
C 6.70378197980585 -17.25236768088886 13.15014308881772
S 7.18740247908070 -16.01286168603983 14.35003167623717
C 8.76282599495343 -16.70651596214567 14.84737374801805
H 5.02408727322586 -16.13031467085558 12.46844975525095
H 4.63729828166010 -17.36714814498337 13.65654754844106
H 6.92600148027952 -18.24188340357465 13.58419753763248
H 7.34591245459082 -17.14490488703804 12.25994140865009
H 8.62950097942090 -17.69028203419499 15.31766865577333
H 9.44139157474874 -16.79450809158877 13.98803072279989
H 9.21021317223951 -16.01962668465959 15.57601117693581
H 5.03054087048864 -19.15861797097130 11.89993635948680
H 5.40836815063368 -17.88909813549878 10.71378732944906
H 3.77562782616956 -17.99561665422454 11.41328930304316
C 11.49083560953115 -20.23949358146374 15.87231947845300
C 11.85789900064437 -19.06373511631520 14.96553606139851
C 13.30134437434539 -19.06239765332062 14.51530314765889
C 14.27480204268472 -18.66611244951812 15.61864679241294
N 15.55450678794911 -18.91107374502786 15.37648211611237
O 13.89138612089285 -18.10392031593094 16.64671691648038
H 11.21484529105190 -19.08043716821927 14.07145350738401
H 11.64332262480266 -18.11488891710007 15.48004117929393
H 13.59234229222455 -20.04001567417617 14.09629078631552
H 13.44694735485086 -18.33216342529378 13.70305350568419
H 15.85969292986372 -19.38838331309961 14.53456188726480
H 16.26114720689009 -18.63472254014065 16.05245589640874
H 12.09509714890427 -20.23995385510964 16.79124727822191
H 11.65957521139915 -21.19889719257995 15.35848603306227
H 10.43190043006602 -20.19823611829144 16.16609899386497
C 7.92730649062296 -15.95286433215384 19.92311006449114
C 7.90795881653854 -14.66871433388953 19.06026872767915
C 9.24851941678557 -14.49348432975990 18.35251745665870
C 6.75935392278874 -14.68229237436053 18.05906667795173
H 7.76284575667294 -13.81009528191526 19.74039224696991

H	9.26277606930824	-13.58404039895138	17.73221486602629
H	10.07810030879700	-14.42548082246399	19.07283393914313
H	9.45188704900370	-15.35185637267594	17.69238794507882
H	6.73587470809942	-13.75524398376285	17.46585216409600
H	6.86311948534068	-15.52291647417216	17.35364013979665
H	5.78572441515560	-14.78640212860095	18.56242103084594
H	8.07571896103002	-16.83944502464966	19.28541480444307
H	6.98048060204837	-16.08291364498055	20.46874893796088
H	8.74549519768158	-15.92278306467085	20.65802878440808
